# Pairwise Correlation Analysis of the Alzheimer’s Disease Neuroimaging Initiative (ADNI) Dataset Reveals Significant Feature Correlation

**DOI:** 10.3390/genes12111661

**Published:** 2021-10-21

**Authors:** Erik D. Huckvale, Matthew W. Hodgman, Brianna B. Greenwood, Devorah O. Stucki, Katrisa M. Ward, Mark T. W. Ebbert, John S. K. Kauwe, The Alzheimer’s Disease Neuroimaging Initiative, The Alzheimer’s Disease Metabolomics Consortium, Justin B. Miller

**Affiliations:** 1 Sanders-Brown Center on Aging, University of Kentucky, Lexington, KY 40536, USA; Erik.Huckvale@uky.edu (E.D.H.); Matthew.Hodgman@uky.edu (M.W.H.); mark.ebbert@uky.edu (M.T.W.E.); 2 Department of Biology, Brigham Young University, Provo, UT 84602, USA; briannabellemathre@gmail.com (B.B.G.); devorah.stucki@gmail.com (D.O.S.); katrisa14@gmail.com (K.M.W.); kauwe@byu.edu (J.S.K.K.)

**Keywords:** ADNI, pairwise feature correlation, feature reduction, machine learning, Alzheimer’s disease

## Abstract

The Alzheimer’s Disease Neuroimaging Initiative (ADNI) contains extensive patient measurements (e.g., magnetic resonance imaging [MRI], biometrics, RNA expression, etc.) from Alzheimer’s disease (AD) cases and controls that have recently been used by machine learning algorithms to evaluate AD onset and progression. While using a variety of biomarkers is essential to AD research, highly correlated input features can significantly decrease machine learning model generalizability and performance. Additionally, redundant features unnecessarily increase computational time and resources necessary to train predictive models. Therefore, we used 49,288 biomarkers and 793,600 extracted MRI features to assess feature correlation within the ADNI dataset to determine the extent to which this issue might impact large scale analyses using these data. We found that 93.457% of biomarkers, 92.549% of the gene expression values, and 100% of MRI features were strongly correlated with at least one other feature in ADNI based on our Bonferroni corrected α (*p*-value ≤ 1.40754 × 10^−13^). We provide a comprehensive mapping of all ADNI biomarkers to highly correlated features within the dataset. Additionally, we show that significant correlation within the ADNI dataset should be resolved before performing bulk data analyses, and we provide recommendations to address these issues. We anticipate that these recommendations and resources will help guide researchers utilizing the ADNI dataset to increase model performance and reduce the cost and complexity of their analyses.

## 1. Introduction

Researchers increasingly leverage big data techniques, such as machine learning, to identify patterns indicative of disease trajectory to better understand, diagnose, and treat Alzheimer’s disease (AD). This search for a cure has led to ever-expanding datasets that have increased in both size and complexity [1]. Although AD is a progressive neurodegenerative disorder characterized by the “A/T/N” system (i.e., β-amyloid biomarker buildup, tau biomarker buildup, and neurodegeneration or neuronal injury) [2], heterogeneity in disease manifestation and trajectory impact our ability to accurately diagnose or treat AD [3,4]. However, since AD is the most common cause of dementia [5], and related AD health-care costs are projected to exceed $1 trillion by 2050 [6], it is imperative to leverage large biobanks to best define its etiology and search for a cure. Here, we utilize the Alzheimer’s Disease Neuroimaging Initiative (ADNI) dataset, which contains patient data for AD cases and controls spanning 49,288 biomarkers and 1.2 terabytes of neuroimages.

While large biological datasets, such as the ADNI cohort, are crucial for developing accurate models, an excessive number of features can cause algorithms to take more time to compute [7,8,9], require significantly more computational resources [9,10,11], increase model complexity [9], reduce model performance [12], and ultimately increase the costs of large-scale analyses. These issues often make these types of analyses intractable for smaller research labs with limited computational resources. Researchers typically sidestep the issue by reducing their analyses to a pre-selected subset of features based on literature searches or specific hypotheses, which limits the creative exploration of other features included in the dataset. Programmatic solutions to feature selection also exist [13] but require a pairwise correlation analysis to identify redundancy [14]. Pairwise correlation analyses iteratively calculate the correlation between each feature and all other feature in the dataset [15]. When multiple features are highly correlated with each other, one feature can be used as representative of all other features, which effectively reduces the size of the dataset for downstream analyses.

We assessed correlation within the ADNI dataset to determine the extent to which machine learning might be impacted by correlated features. We performed a pairwise correlation analysis of all 49,288 biomarkers and 793,600 extracted magnetic resonance imaging (MRI) features (842,888 total features). We repeated the pairwise correlation analysis using subsets stratified by sex and clinical dementia rating (CDR) to determine if the correlated features should be interpreted broadly (i.e., across the dataset) or more narrowly (e.g., only in females). We identified high feature redundancy that impacts 99.566% of all features, including 93.457% of the ADNIMERGE features and 92.549% of the gene expression features. Additionally, we identified metadata in the ADNI tables that were not programmatically distinguishable from biomarkers, and several duplicate features with different column headers.

We propose that machine learning on the ADNI dataset should remove highly correlated or duplicate features and metadata to increase model performance, decrease model training time, and accelerate AD research toward improved understanding, diagnosis, and treatment. We provide correlation tables to facilitate the identification and filtering of highly correlated features within ADNI.

## 2. Materials and Methods

Data used in the preparation of this article were obtained from the Alzheimer’s Disease Neuroimaging Initiative (ADNI) database (adni.loni.usc.edu) on 15 November 2019. The ADNI was launched in 2003 as a public-private partnership, led by principal investigator Michael W. Weiner, MD. The primary goal of ADNI has been to test whether serial magnetic resonance imaging (MRI), positron emission tomography (PET), other biological markers, and clinical and neuropsychological assessment can be combined to measure the progression of mild cognitive impairment (MCI) and early Alzheimer’s disease (AD). For up-to-date information, see www.adni-info.org. ADNI researchers collect, validate, and utilize data, including MRI and PET images, genetics, cognitive tests, CSF and blood biomarkers as predictors of the disease.

We divided the ADNI data into three domains: the ADNIMERGE domain, which contains features such as cerebral spinal fluid (CSF) biomarkers and cognitive function test scores; the gene expression domain, which contains gene expression levels from blood microarrays [16]; and the MRI domain, which contains features we extracted from MRIs using deep convolutional autoencoders. Step-by-step protocols for each domain are included at https://github.com/jmillerlab/ADNI_Correlation and described below.

### 2.1. ADNIMERGE Domain

We constructed the ADNIMERGE domain using the R package, ADNIMERGE [17]. We retrieved the data from ADNIMERGE because it contains the ADNI tabular data in the form of multiple individual tables conveniently stored within a single package. To efficiently merge these data, we developed a custom method for combining the ADNIMERGE tables into one table by joining each table by its patient ID and most recent measurement (see Figure S1).

We preprocessed all tables in the ADNIMERGE domain before combining them. We capitalized all headers to have consistent feature names across tables with overlapping features. Columns with only one unique value were removed because features without variation are uninformative in machine learning. Every feature table contained patient IDs that we used as primary keys when combining tables. We removed the ‘Data Dictionary’ table from the domain because it contained only meta-data and no patient IDs. We recorded the data type of each feature, whether nominal or numeric, to determine which statistical tests to apply in downstream analysis. All features containing number values were marked as numeric unless they contained fewer than ten unique values, in which case they were considered nominal. Features containing text were marked as nominal. However, if those values contained more than 20 unique values, they were removed from the ADNIMERGE domain to eliminate features that were unique or almost unique for the individual, which might occur when the features are unique identifiers or notes written by the data recorders.

We further cleaned the data so that every feature had a single value for each individual. For features that contained longitudinal data, we selected the most recent value using its recording date. If the recording dates were not available, we arbitrarily selected one value for the person. If an individual did not have a value for a certain feature, we marked it as unknown. We removed features that either contained only unknown values or only one unique value because those features are uninformative in machine learning. The resulting table contained rows corresponding to each person, and columns corresponding to each ADNIMERGE feature.

Lastly, we resolved unknown values by either removing or imputing them. Features with fewer than 80% known values were removed to ensure accuracy. Nominal features with fewer than 20 patients in any of their categories were also removed as these features did not meet the assumptions of our statistical tests. Numeric values were then imputed using a Bayesian-ridge estimator [18] that predicts unknown values for numeric features based on known values of other features. The random number generator for this stochastic algorithm was seeded for reproducibility. A simple imputer was used for unknown nominal values, which replaced unknown values with the most frequent known category. These imputing algorithms were provided by the Scikit-learn Python package [19]. The completed ADNIMERGE data set contained 1131 features.

### 2.2. Gene Expression Domain

We downloaded the gene expression domain from ADNI, which contains a table of gene expression profiles from blood RNA and has previously been explored using machine learning [16]. All quality control and normalization were conducted by ADNI before its inclusion in the dataset. We transposed the table so that feature columns corresponded to the normalized gene expression levels for each patient. Any columns that contained metadata or did not contain a header were removed. The resulting gene expression domain contained a total of 48,157 genes.

### 2.3. MRI Domain

The MRI domain contained features we extracted from MRIs using deep convolutional autoencoders designed and trained using the PyTorch deep learning library [20]. All features in this dataset were numeric transformed pixel values. The image dataset initially consisted of 1.2 terabytes of MRIs, but we used only MRIs that belonged to the 743 patients also found in both the ADNIMERGE and gene expression domains. We organized these MRIs using the PyDicom Python package [21] so that each patient had a sequence of MRI images scanned from one side of the skull to the other. We used the med2image [22] Python package to convert the MRIs from DICOM format to PNG so that they could be used in deep learning. All images were resized to 128 by 128 pixels using the OpenCV Please confirm that the intended meaning has been retained.ython package [23]. Image pixel values were then normalized using min-max normalization [24] to optimize them for the deep learning model.

**Figure 1:**
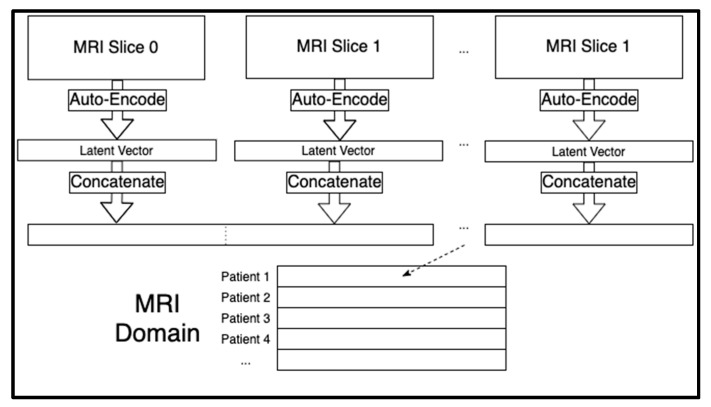
Creation of the MRI domain from the MRI slice sequences using the trained convolutional autoencoders. A separate autoencoder was trained for each MRI slice, and the latent space was concatenated for each person to create a row specific to that individual.

Each patient had a sequence of 124 sagittal MRI slices. Each of those two-dimensional images were compressed to a one-dimensional latent space of 6,400 extracted features. By storing images in one-dimensional arrays, the MRI domain could be tabular and therefore merged with the other two domains. We trained separate autoencoders for each of the 124 slice indices of the MRI sequences across all the patients using the Adam optimizer for artificial neural networks [25] (See Figure S2). The 124 latent vectors for each individual were concatenated for a total of 124 × 6400 = 793,600 extracted MRI features per person. These concatenated MRI features acted as the rows in the MRI domain (see Figure 1). We seeded all random number generators for reproducibility since the model training algorithms are stochastic in nature.

### 2.4. Combining All Domains

We merged the ADNIMERGE, gene expression, and MRI domains into a singular dataset that we used for our correlation analysis. The combined dataset contained a total of 1131 ADNIMERGE features + 48,157 gene features + 793,600 MRI features = 842,888 features for 743 individuals.

### 2.5. Correlation Analysis

We performed a pairwise correlation analysis where we compared every feature in our dataset to every other feature. For each comparison, we chose a statistical test depending on the data types of the two features as well as the normality of their distribution, if numeric (See Table 1).

The statistical tests and the test for normality [26] were conducted using the SciPy Python package [27]. Because we analyzed all pairwise comparisons (excluding self-comparisons) of *m* features across *n* individuals, the big-O time complexity of our algorithm was *O*(*n* ∗ *m*^2^). However, we optimized performance by using parallel processing across four processing cores. Because of the high number of comparisons (355,229,668,828), we employed a Bonferroni corrected α value of α = 1.40754 × 10^−13^ (0.05/355,229,668,828). Only feature comparisons with significant *p*-values were stored to save disk space.

### 2.6. Subset Stratification Analysis

Feature correlation within the entire dataset may occur if a subset of individuals determine that correlation. Therefore, we determined if the significantly correlated features were also correlated in different subsets stratified by sex (e.g., male or female) and clinical dementia rating (CDR; e.g., 0, 0.5, ≥1) [28]. Sex-specific AD pathologies occur [29], and pathologies vary based on cognitive status [29]. Additionally, if disease-modifying treatments in AD cases affect feature correlation, the features would not be correlated in all subsets. Therefore, if the features remain correlated in the complete dataset and each stratified subtype, they can be considered redundant.

We created five subsets from the combined dataset: female patients, male patients, cognitive normal controls where CDR = 0, patients with mild cognitive impairment where CDR = 0.5, and patients with AD where CDR ≥ 1.0. Next, we identified correlations with the highest possible significance (*p*-value ≤ 5 × 10^−324^, which is the smallest positive value in Python 3.7) from the original analysis of the combined dataset. We reran those correlation analyses within each of the five subsets to determine if certain stratifications affected the correlation significance. We noted that significance will drop due to smaller sample size in the comparison and some features were dropped from the analysis (e.g., features with only one unique value in the subset). In practice, only the AD subset experienced loss of significant comparisons as a result of sub-setting.

## 3. Results

We found that 839,226 ADNI features (99.566% of the total number of features) are significantly correlated with at least one other feature (93.457% of the ADNIMERGE features, 92.549% of the gene expression features, and 100% of the MRI features). Table 2 shows a subset of features that are correlated at the highest significance threshold with more than one other feature, including patient sex, intra-cranial volume, various neuropsychological batteries, ventral diencephalon volume, and cerebrospinal fluid (CSF) glucose levels.

While Table 2 shows the numbers of correlated features for seven example features from the ADNIMERGE domain, Table S1 shows the same but for all the ADNIMERGE features. Both Table 2 and Table S1 show the numbers of correlated features based on our Bonferroni corrected α (*p*-value ≤ 1.40754 × 10^−13^). However, Table S2 shows the numbers of correlated features based on the maximally significant α (*p*-value ≤ 5 × 10^−324^). The complete table (gene expression and MRI features included in addition to ADNIMERGE) for the Bonferroni corrected α is available online at: https://github.com/jmillerlab/ADNI_Correlation/blob/main/data/sig-freqs/bonferroni-sig-freqs.csv.

The complete table for the maximally significant α is available online at: https://github.com/jmillerlab/ADNI_Correlation/blob/main/data/sig-freqs/maximum-sig-freqs.csv.

While the complete tables containing our results are available online, we provide a summary of those results in Table 3 (Bonferroni corrected α) and Table 4 (maximally significant α).

### 3.1. Domain-Specific Correlation Analysis Results

First, we report the number of times ADNIMERGE features correlated with each domain-specific feature in the dataset, after correcting for multiple testing using a Bonferroni α value (Table 3A). Although many gene expression probes were not highly correlated with ADNIMERGE features (mean = 0.28 ± 5.52), gene expression for ubiquitin specific peptidase 9 Y-linked (*USP9Y*; probe set: 11725293_at) was correlated with the 189 ADNIMERGE features. In contrast, many ADNIMERGE features were highly correlated with other ADNIMERGE features (mean = 129.49 ± 88.06 highly correlated comparisons per feature). The volume (cortical parcellation) of right fusiform (ADNIMERGE header: ST85CV) was correlated with 346 ADNIMERGE features, which was the highest frequency. Similarly, MRI features displayed high correlation with many ADNIMERGE data (mean = 9.31 ± 20.09), and one MRI feature was highly correlated with 188 ADNIMERGE features.

Next, we report how many gene expression features correlate with each domain-specific feature using a Bonferroni corrected α (Table 3). For ADNIMERGE features, the patient date of birth correlates with the most Affymetrix probes (616 gene probes), and the mean number of significant comparisons per feature was 11.91 ± 30.9. The MRI dataset also contained many significant correlations (mean = 7.87 ± 19.66), and one feature from the MRI autoencoder correlated with 149 gene expression values. Gene expression features, on average, strongly correlated with 6139.72 ± 6195.45 other gene expression probes. The probe expression levels for adducin 2 (*ADD3*; probe set: 11721606_a_at) are strongly correlated with the most other gene probes (24,588 probes), which consists of 51.058% of the total number of probes in the dataset.

Finally, we report the number of significant correlations with the MRI-extracted features from the autoencoder (Table 3). A single gene expression probe for the X-inactive specific transcript (*XIST*) non-protein coding region (probe set: 11757857_s_at) was strongly correlated with 119,556 MRI features, and gene probes, on average, were highly correlated with 140.05 ± 3642.09 MRI features. The ADNIMERGE features were correlated with 6988.04 ± 19,170.23 MRI features and patient sex had the highest number of significant correlations with MRI features (145,780 significant correlations). MRI to MRI feature redundancy is even higher, with a single MRI feature being correlated with 347,944 other MRI features, and each MRI feature had significant feature redundancy (minimum = 81; mean = 141,348.57 ± 69,866.96).

Similar analyses were conducted on the total frequencies of feature redundancy (Table 3) and using the maximum significance threshold (Table 4).

### 3.2. Subset Stratification Analysis Results

Tables S3–S7 contain the summary statistics for the five subsets. Differences in these tables compared to Table 4 indicate that comparisons that were maximally significant using the entire data set (all 743 patients) were not maximally significant for a given subset. We found that the male, female, and AD subsets each exhibited fewer maximally significant feature comparisons than found in the complete correlation analysis (compare Tables S3, S4, and S7 with Table 4). Features lost an average of 1391.485 ± 2835.093 features that they were maximally correlated with (i.e., based on the maximally significant α of 5 × 10^−324^) after correlation analysis on the stratified male subset. Likewise, features lost an average of 1323.918 ± 2694.103 maximally significant correlations after analysis on the female subset. Finally, an average of 2343.442 ± 4325.865 correlations per feature were lost using the AD subset. Conversely, for the healthy control and mild cognitive impairment subsets, all feature comparisons maintained maximum significance (compare Tables S5 and S6 to Table 4). While it is a small amount, some of the loss in the AD subset is attributed to the sub-setting itself due to features in the subset no longer having more than one unique value or no longer satisfying the assumptions of our statistical tests. Tables S8–S12 (where each table represents a different subset) show that the sub-setting alone resulted in no loss of maximally significant comparisons except in the AD subset since the AD subset table is the only one with values that differ from Table 4. Specifically, features lost an average of 2.643 ± 0.005 correlations due to the AD subset stratification alone (i.e., the small sample size resulted in fewer possible correlation comparisons).

### 3.3. Feature-Correlation Mappings

We created a mapping from each gene expression and ADNIMERGE feature to a list of gene expression and ADNIMERGE features with which they are strongly correlated (based on the Bonferroni corrected α). Table S13 shows the computational resources used to conduct these comparisons. There were 1,214,628,828 comparisons (0.342% of all comparisons) that involved only ADNIMERGE features or gene expression features and our mapping took up 1.87 gigabytes of disk space. We excluded MRI features from the mapping because autoencoder features do not have clear biological significance and filesharing size restraints would preclude including those comparisons online (~550 gigabytes of disk space). The ADNIMERGE and gene mappings are available as a downloadable Python pickle file. We chose the pickle file format because it facilitates easy integration with Python scripts and research pipelines. This file is available online at: https://drive.google.com/file/d/1uRuT6rhDVDeeBuRYPif3Ate3u1UVs-hO/view?usp=sharing.

## 4. Discussion

Our results demonstrate a high amount of feature redundancy in the ADNI dataset that should be considered when using the dataset for machine learning. While we make no claims about feature correlation in other large-scale databanks, the significant feature correlation in ADNI suggests that this issue might be more widespread than previously thought and should be considered before performing large-scale data mining. For example, a single gene expression feature alone could replace more than half the gene expression values because it is highly correlated with expression in each of those genes. Thousands of MRI features can be replaced by ADNIMERGE or gene expression features, and the MRI features themselves can be further reduced. A single MRI feature can replace up to 43.844% of the MRI domain, indicating that we could almost double the MRI compression ratio. This redundancy may inhibit the types of analyses possible in research laboratories with limited computational resources. Furthermore, laboratories using the ADNI features for large-scale data analyses are likely to waste computational time and resources if they do not properly deal with feature redundancy within the dataset. Beyond that, models analyzing redundant ADNI data are expected to perform and generalize poorly because of the curses of dimensionality [30] and overfitting [31]. To help alleviate these issues, we provided future researchers with a mapping of highly correlated features within the ADNIMERGE and gene expression domains. We recommend that researchers using the ADNIMERGE and gene expression data download our mapping file, and we inform researchers using the MRI features of their high redundancy. Future work can include further reducing the size of the MRI domain using a 1D autoencoder, as compared to the 2D autoencoder we used to perform the initial reduction. This architectural change would likely be useful because while 2D convolutional autoencoders compress 2-dimensional data (e.g., MRIs), 1D convolutional autoencoders compress 1-dimensional data (e.g., our extracted MRI features).

Another benefit of our correlation analysis is that it shows the possibility of AD-related features, which are more costly to collect, being replaced by less costly features. For example, collecting CSF biomarkers requires intrusive lumbar punctures [32], and certain cognitive tests (i.e., Mini-Mental State Examination, Alzheimer’s Disease Assessment Scale-Cognitive Subscale, Frontal Assessment Battery, etc.) are time-intensive and can be stressful for patients [33]. If such non-ideal features are strongly correlated with more palatable features, they can be replaced by data that is easier to collect. Gene expression and MRI features are strongly correlated with hundreds of ADNIMERGE features. There are varying costs in obtaining these ADNIMERGE features (e.g., time, emotional toll, and money). If such biomarkers or tests were more demanding than a relatively simple blood [14] test or MRI scan, they could be replaced by other highly correlated features that are less costly. Doing so may decrease the burden on both patients and caretakers by limiting the number of tests performed or surveys taken, as well as the amount of paperwork that needs to be completed. Additionally, this knowledge may decrease the overall costs of conducting a clinical trial or establishing a cohort if a specific test is no longer required because it does not provide additional data beyond other testing.

Furthermore, our analyses revealed issues with the ADNI data that obstruct data analysis. First, there were several features we discovered to be highly correlated with others but were merely meta-data. These features include the date a measurement was recorded, the version of ADNI when the measurement took place, or identification numbers such as bar-code, sample-identification, lonis ID, image UID etc. While these features serve an important function in the data, they do not have biological or cognitive meaning. We recommend that such features are labeled and distinguished, so that computational researchers can programmatically identify them in their scripts and separate them from the rest of the data. We recommend the same for columns in ADNI tables that appear to be notes taken by the data recorders. ADNIMERGE could include a two-column table that maps features to their designation (e.g., metadata, biomarker, MRI, etc.).

Another issue we identified was that certain features were maximally correlated with other features that had only slight deviations in their names and are likely duplicates in the dataset. For example, Table 5 shows that two features representing intracranial volume had exactly equal results but different header names.

We suspect that such features are equivalent, but they have different header names when appearing in two different tables. If multiple tables contain columns with the same features, we recommend that such columns are correctly labeled by having the exact same header name across all tables in which they appear.

We recognize that the ADNI dataset contains longitudinal data that may result in different levels of feature correlation at different time points. We chose the last recorded time series datapoint for each feature to ensure that analyzed features were collected at similar points in disease progression. Since we conducted subtype analyses that show no difference in correlation between sex or cognitive decline, feature correlation can be interpreted broadly at the population level. However, we recognize that certain limitations in the ADNI dataset (e.g., age at first patient measurement, incomplete time series data, imputation, etc.) may limit our ability to detect changes in feature correlation for individuals. Additionally, deviations from feature correlation across a time series for an individual may warrant further investigation for its disease association.

The loss of significant statistical tests performed on the subsets may be partially a result of the reduced sample size. Patients with AD represent the smallest subset, therefore having the lowest statistical power, which may explain why the AD subset had the largest drop in significant tests. However, neither the healthy control subset nor the mild cognitive impairment subset experienced any loss of significant comparisons despite being smaller than the male subset, which did lose significant comparisons. This retention of comparison frequencies suggests that other factors beyond sample size contribute to the loss of statistically significant comparisons and that some feature comparisons lose significance when stratified by sex. Similarly, our results show that comparisons become less significant when stratified by CDR and performed on the AD subset, which does not occur in the control or mild cognitive impairment subsets. The reduction in features that are testable in each subset contributes slightly to this reduction in significant comparisons but does not account for all differences (see Table S12). Therefore, not all strongly correlated feature pairs remain correlated in each CDR subset.

## 5. Conclusions

Our analyses contribute significantly to future AD research by exploring feature correlation within the ADNI dataset. We identified many non-ic ADNI features that are highly correlated with each other and can be replaced when building large data models. We provide a template for constructing a convolutional autoencoder capable of extracting tabular features from MRIs and inform future researchers of the redundancy among these MRI features. Additionally, we propose solutions to address feature redundancy within the non-MRI features by downloading our feature redundancy tables. We validated these correlations by sub-setting the ADNI dataset and found that most highly correlated features remain highly correlated in each stratified subset. Additionally, we propose that researchers who design clinical trials or testing for AD should be mindful of feature redundancy to reduce the unnecessary testing burden on patients and caregivers when the tests do not elicit additional information. We anticipate that this research will help guide researchers using machine learning on the ADNI dataset to take into account feature redundancy in the future.

## Figures and Tables

**Table 1:** Statistical tests chosen for comparisons and their conditions.

Comparison Data Types	Condition	Statistical Test
Numeric and Numeric	Both features follow a normal distribution	Pearson correlation
Numeric and Numeric	At least one of the features does not follow a normal distribution	Spearman correlation
Categorical and Categorical	The contingency table contains at least one frequency less than five	N/A
Categorical and Categorical	All frequencies in the contingency table are greater than or equal to five	Chi-squared
Numeric and Categorical	All categories have a normal distribution	ANOVA
Numeric and Categorical	Not all categories have a normal distribution	Kruskal-Wallis

For numeric features, depending on the normality of their distribution, we chose between a parametric (normal distribution) or a non-parametric (non-normal distribution) statistical test. If both features were nominal, we used the Chi-squared test unless the contingency table resulting from the two features did not each contain at least five instances. In that case, the test was not performed.

**Table 2:** ADNIMERGE Features that are Highly Correlated with other Features.

Feature Name	ADNIMERGE Frequency	Gene Expression Frequency	MRI Frequency	Total Frequency
PTGENDER	207	281	145,780	146268
ICV	265	84	143,377	143,724
CLOCKNUM	199	0	97,725	97,924
COPYTIME	243	0	97,030	97,228
CLOCKSYM	216	0	96,307	96,523
ST65SV	191	46	81,250	81,487
GLUCOSE	155	0	81,245	81,400

Numbers of correlated features for seven example features in the ADNIMERGE domain. The ‘Feature Name’ is the column header as it appeared in our constructed tabular data set. The ‘ADNIMERGE Frequency’ is the number of ADNIMERGE features that are highly correlated with the feature. For example, intra-cranial volume (ICV) is correlated with 265 other ADNIMERGE features. It is likewise correlated with 84 gene expression levels and 143,377 extracted MRI features. The ‘Total Frequency’ is the sum of the ‘ADNIMERGE Frequency’, ‘Gene Expression Frequency’, and ‘MRI Frequency’. In other words, it is the total number of features that are highly correlated with each row across the entire ADNI data set.

**Table 3:** Summarized correlated feature frequencies based on the Bonferroni corrected α.

**A—ADNIMERGE Frequencies**					**B—Gene Expression Frequencies**				
**Domain**	**Average**	**Standard Deviation**	**Minimum**	**Maximum**	**Domain**	**Average**	**Standard Deviation**	**Minimum**	**Maximum**
ADNIMERGE	129.49	88.06	1	346	ADNIMERGE	11.91	30.9	0	616
Gene Expression	0.28	5.52	0	189	Gene Expression	6139.72	6195.45	1	24,588
MRI	9.31	20.09	0	188	MRI	7.87	19.66	0	149
**C—MRI Frequencies**					**D—Total Frequencies**				
**Domain**	**Average**	**Standard Deviation**	**Minimum**	**Maximum**	**Domain**	**Average**	**Standard Deviation**	**Minimum**	**Maximum**
ADNIMERGE	6988.04	19,170.23	0	145,780	ADNIMERGE	7129.43	19,203.93	1	146,268
Gene Expression	140.05	3642.09	0	119,556	Gene Expression	6280.05	7096.48	1	120,141
MRI	141,348.57	69,866.96	81	347,944	MRI	141,365.75	69,873.31	81	347,955

Summary of the numbers of correlated features based on the Bonferroni corrected α. Sections A through D provide summary statistics for the domain frequencies for ADNIMERGE, Gene Expression, MRI, and Total. For example, the meaning of the ‘Average’ column and ‘MRI’ row in table A is the average number of ADNIMERGE features with which the MRI features are strongly correlated. That row states that the MRI features are strongly correlated with an average of 9.31 ADNIMERGE features with a standard deviation of 20.9 features. The 0 in the ‘Minimum’ column indicates that at least one MRI feature is not correlated with any ADNIMERGE features. The 188 under ‘Maximum’ indicates that at least one MRI feature is correlated with 188 ADNIMERGE features when *p*-value ≤ 1.40754 × 10^−13.^

**Table 4:** Summarized correlated feature frequencies based on the maximally significant α.

**A—ADNIMERGE Frequencies**					**B—Gene Expression Frequencies**				
**Domain**	**Average**	**Standard Deviation**	**Minimum**	**Maximum**	**Domain**	**Average**	**Standard Deviation**	**Minimum**	**Maximum**
ADNIMERGE	5.48	5.83	1	23	ADNIMERGE	0.0	0.0	0	0
Gene Expression	0.0	0.0	0	0	Gene Expression	1.55	0.94	1	7
MRI	0.0	0.0	0	0	MRI	0.0	0.0	0	0
**C—MRI Frequencies**					**D—Total Frequencies**				
**Domain**	**Average**	**Standard Deviation**	**Minimum**	**Maximum**	**Domain**	**Average**	**Standard Deviation**	**Minimum**	**Maximum**
ADNIMERGE	0.0	0.0	0	0	ADNIMERGE	5.48	5.83	1	23
Gene Expression	0.0	0.0	0	0	Gene Expression	1.55	0.94	1	7
MRI	2457.08	4397.99	1	11957	MRI	2457.08	4397.99	1	11,957

Summary of the numbers of correlated features based on the maximally significant comparisons (*p*-value ≤ 5 × 10^−324^). Interestingly, when applying a maximally significant α, features were only strongly correlated with other features in their same domain.

**Table 5:** Example of Features with Identical Results but Slightly Different Names.

Feature	ADNIMERGE Frequency	Gene Expression Frequency	MRI Frequency	Total Frequency	Domain
ICV	265	84	141,676	142,025	ADNIMERGE
ICV.BL	265	84	141,676	142,025	ADNIMERGE

## Data Availability

All custom scripts for processing and analyzing our data are available online at: https://github.com/jmillerlab/ADNI_Correlation. The tables containing the numbers of correlated features for each feature in our constructed data set are available online at: Bonferroni corrected α: https://github.com/jmillerlab/ADNI_Correlation/blob/main/data/sig-freqs/bonferroni-sig-freqs.csv. Maximally significant α: https://github.com/jmillerlab/ADNI_Correlation/blob/main/data/sig-freqs/maximum-sig-freqs.csv. The pickle file containing the mapping of non-MRI features to the non-MRI features that they are correlated with is available online at: https://drive.google.com/file/d/1uRuT6rhDVDeeBuRYPif3Ate3u1UVs-hO/view?usp=sharing.

## Supplementary Materials

The following are available online at https://www.mdpi.com/article/10.3390/genes12111661/s1, Supplementary Figures: Figure S1: Steps for Creating the Raw Data Set for the ADNIMERGE Domain; Figure S2: Diagram Displaying the Creation of the Convolutional Autoencoders. Supplementary Tables: Table S1: Significant Comparison Frequencies For Features By Bonferroni Alpha (ADNIMERGE Features Only); Table S2: Significant Comparison Frequencies For Features By Maximum Alpha (ADNIMERGE Features Only); Table S3: Comparison Frequencies of The Subset Analysis (Male Subset); Table S4: Comparison Frequencies Of The Subset Analysis (Female Subset); Table S5: Comparison Frequencies Of The Subset Analysis (CDR = 0 Subset); Table S6: Comparison Frequencies Of The Subset Analysis (CDR = 0.5 Subset); Table S7: Comparison Frequencies Of The Subset Analysis (CDR ≥ 1.0 Subset); Table S8: Maximally-significant correlations with sufficient data to perform subsetting: Males; Table S9: Maximally-significant correlations with sufficient data to perform subsetting: Females; Table S10: Maximally-significant correlations with sufficient data to perform subsetting: CDR = 0; Table S11: Maximally-significant correlations with sufficient data to perform subsetting: CDR = 0.5; Table S12: Maximally-significant correlations with sufficient data to perform subsetting: CDR ≥ 1.0; Table S13: Resource Usage.
