# Supplementary material for: Pairwise Correlation Analysis of the Alzheimer’s Disease Neuroimaging Initiative (ADNI) Dataset Reveals Significant Feature Correlation
Source: Genes (Basel). 2021 Oct 21;12(11):1661. doi: 10.3390/genes12111661 (PMC8619902; doi:10.3390/genes12111661)
Supplement: Supplementary file 1 [file genes-12-01661-s001.zip › genes-1418687-supplementary.pdf]

Supplemental Material: Pairwise Correlation Analysis of the Alzheimer's Disease Neuroimaging Initiative (ADNI) Dataset Reveals Significant Feature Correlation

Erik D. Huckvale[1], Matthew W. Hodgman[1], Brianna B. Greenwood[2], Devorah O. Stucki[2], Katrisa Ward[2], Mark T.W. Ebbert[1], John S.K. Kauwe[2], for the Alzheimer's Disease Neuroimaging Initiative[+], Justin B. Miller[1,*],

[1]Sanders-Brown Center on Aging, University of Kentucky, Lexington, KY 40536, USA

[2]Department of Biology, Brigham Young University, Provo, UT 84602, USA

[*]Corresponding author: justin.miller@uky.edu

[+]Data used in preparation of this article were obtained from the Alzheimer’s Disease Neuroimaging Initiative (ADNI) database (adni.loni.usc.edu). As such, the investigators within the ADNI contributed to the design and implementation of ADNI and/or provided data but did not participate in analysis or writing of this report. A complete listing of ADNI investigators can be found at: http://adni.loni.usc.edu/wp-content/uploads/how_to_apply/ADNI_Acknowledgement_List.pdf

Table of Contents

Supplementary Figures *3*

Figure S1 – Steps for Creating The Raw Data Set for the ADNIMERGE Domain........3

Figure S2 – Diagram Displaying the Creation of the Convolutional Autoencoders........4

Supplementary Tables *5*

Table S1 – Significant Comparison Frequencies For Features By Bonferroni Alpha (ADNIMERGE Features Only)........5

Table S2 – Significant Comparison Frequencies For Features By Maximum Alpha (ADNIMERGE Features Only).......35

Table S3: Comparison Frequencies Of The Subset Analysis (Male Subset) 44

Table S4: Comparison Frequencies Of The Subset Analysis (Female Subset) 44

Table S5: Comparison Frequencies Of The Subset Analysis (CDR=0 Subset) 45

Table S6: Comparison Frequencies Of The Subset Analysis (CDR=0.5 Subset) 45

Table S7: Comparison Frequencies Of The Subset Analysis (CDR≥1.0 Subset) 46

Table S8– Maximally-significant correlations with sufficient data to perform subsetting: Males........46

Table S9– Maximally-significant correlations with sufficient data to perform subsetting: Females 46

Table S10– Maximally-significant correlations with sufficient data to perform subsetting: CDR=0 47

Table S11– Maximally-significant correlations with sufficient data to perform subsetting: CDR=0.5 47

Table S12– Maximally-significant correlations with sufficient data to perform subsetting: CDR≥1.0 47

Table S13 – Resource Usage 48

Supplementary Figures

Figure S1 – Steps for Creating The Raw Data Set for the ADNIMERGE Domain

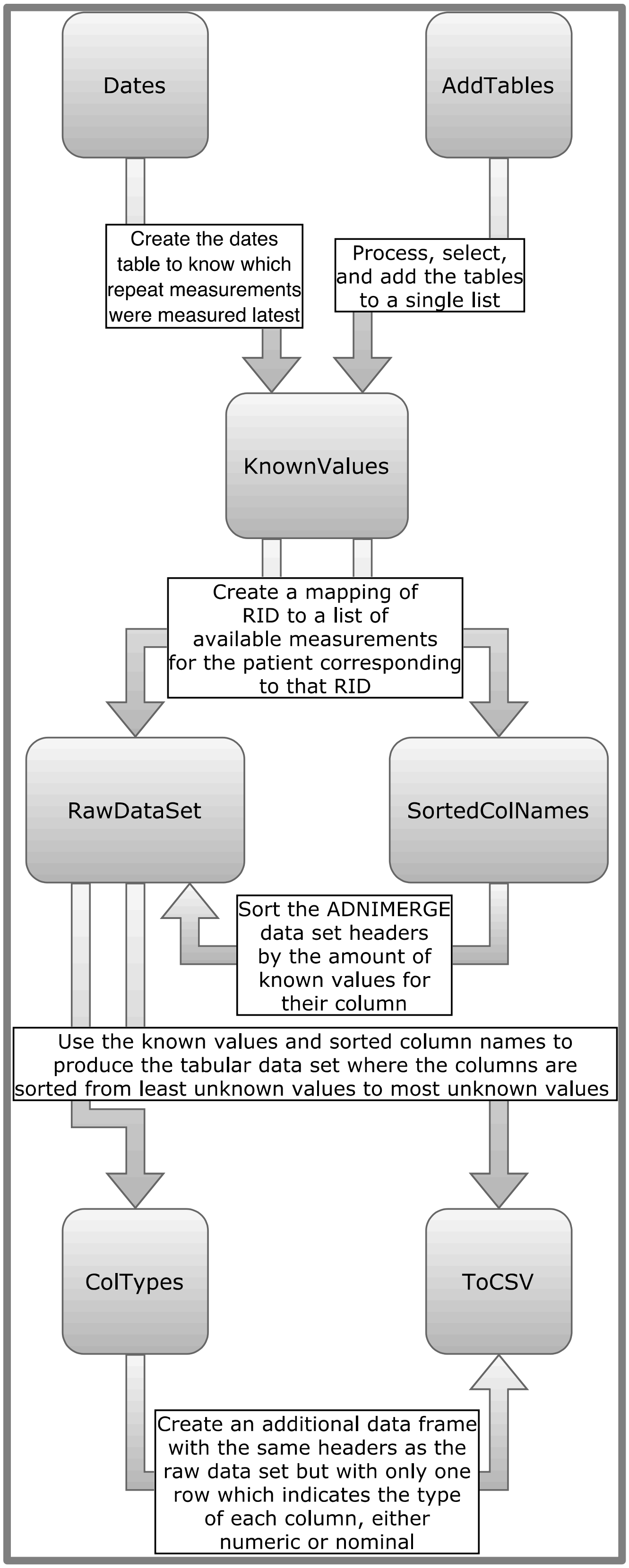

Figure S2 – Diagram Displaying the Creation of the Convolutional Autoencoders

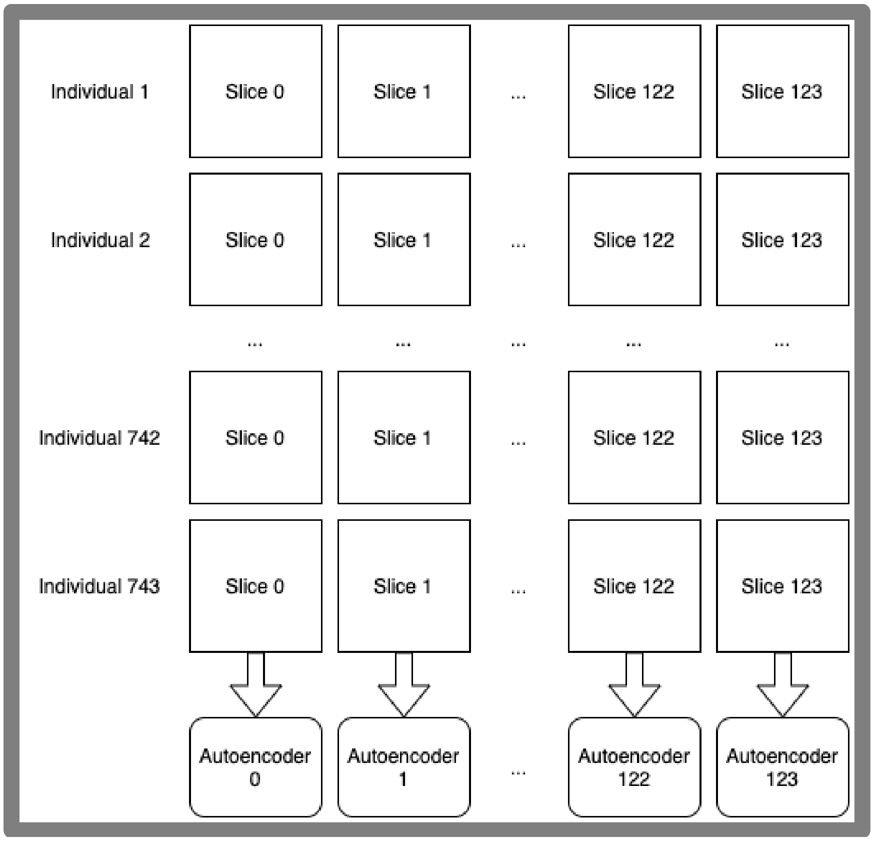

Supplementary Tables

Table S1 – Significant Comparison Frequencies For Features By Bonferroni Alpha (ADNIMERGE Features Only)

Feature	ADNIMERGE Frequency	Gene Expression Frequency	MRI Frequency	Total Frequency	Domain
PTGENDER	207	281	145780	146268	ADNIMERGE
ST10CV	262	85	143377	143724	ADNIMERGE
ICV	265	84	141676	142025	ADNIMERGE
ICV.BL	265	84	141676	142025	ADNIMERGE
QCTYPE	213	0	114599	114812	ADNIMERGE
FSVERSION	213	0	114599	114812	ADNIMERGE
EVENT	209	0	109326	109535	ADNIMERGE
INCLUSION	208	0	108130	108338	ADNIMERGE
COLPROT	215	3	107022	107240	ADNIMERGE
ORIGPROT	215	3	107022	107240	ADNIMERGE
ENROLLED	215	3	107022	107240	ADNIMERGE
CLOCKNUM	199	0	97725	97924	ADNIMERGE
COPYTIME	198	0	97030	97228	ADNIMERGE
CLOCKSYM	216	0	96307	96523	ADNIMERGE
CLOCKTIME	243	0	96043	96286	ADNIMERGE
ST65SV	191	46	81250	81487	ADNIMERGE
GLUCOSE	155	0	81245	81400	ADNIMERGE
BRAINVOL	274	58	80547	80879	ADNIMERGE
USERDATE	196	0	80393	80589	ADNIMERGE
ARM	339	0	78446	78785	ADNIMERGE
ILE	78	60	76698	76836	ADNIMERGE
C13.PE.P.16.0.22.6	153	0	76085	76238	ADNIMERGE
C13.PE.16.0.22.6	154	0	74416	74570	ADNIMERGE
ST35SA	156	60	73101	73317	ADNIMERGE
ST94SA	152	56	71937	72145	ADNIMERGE
ST56SA	246	59	69892	70197	ADNIMERGE
VSWEIGHT	34	55	68231	68320	ADNIMERGE
ST110SA	220	72	66809	67101	ADNIMERGE
ST58SA	197	55	63784	64036	ADNIMERGE
ST51SA	216	68	61998	62282	ADNIMERGE
ST117SA	219	53	61404	61676	ADNIMERGE
WHOLEBRAIN.BL	279	60	60265	60604	ADNIMERGE
IMAGEUID	155	4	60270	60429	ADNIMERGE
IMAGEUID.BL	155	4	60270	60429	ADNIMERGE
WHOLEBRAIN	279	60	60015	60354	ADNIMERGE
ST49SA	226	57	58630	58913	ADNIMERGE

IMAGEUID_2	161	9	58717	58887	ADNIMERGE
IMAGEUID_1	158	5	57163	57326	ADNIMERGE
LONISID	156	7	55744	55907	ADNIMERGE
ST80SV	125	77	55705	55907	ADNIMERGE
PTNO	157	7	55716	55880	ADNIMERGE
ST40SA	197	51	55574	55822	ADNIMERGE
EXAMDATE.BL	152	7	54576	54735	ADNIMERGE
ST111SA	230	58	54263	54551	ADNIMERGE
ST108SA	226	56	53499	53781	ADNIMERGE
ST124SV	203	42	53000	53245	ADNIMERGE
ST115SA	233	57	52643	52933	ADNIMERGE
TAURINE	129	0	52713	52842	ADNIMERGE
ST52SA	206	52	51936	52194	ADNIMERGE
SAMPLE_BAR_CODE	165	8	51969	52142	ADNIMERGE
VAL	77	1	51265	51343	ADNIMERGE
LEFT_CEREBRAL_WHITE_MATTER_VOLUME	257	50	50790	51097	ADNIMERGE
ST99SA	240	52	50566	50858	ADNIMERGE
SAMPLE.BAR.CODE	163	7	50321	50491	ADNIMERGE
RIGHT_CEREBRAL_WHITE_MATTER_VOLUME	255	50	49763	50068	ADNIMERGE
ST90SA	228	60	49349	49637	ADNIMERGE
URNVOL	151	0	49357	49508	ADNIMERGE
URN	149	0	49301	49450	ADNIMERGE
SAMPLE.IDENTIFICATION	156	6	48127	48289	ADNIMERGE
ST82SA	120	56	47444	47620	ADNIMERGE
VENTRICLE_3RD_VOLUME	134	74	46036	46244	ADNIMERGE
RIGHT_CHOROID_PLEXUS_VOLUME	139	71	45453	45663	ADNIMERGE
ST105SA	132	53	43618	43803	ADNIMERGE
ST120SV	250	43	41733	42026	ADNIMERGE
ST114SA	238	58	40147	40443	ADNIMERGE
ST109SA	180	25	38576	38781	ADNIMERGE
ST36SA	236	52	38176	38464	ADNIMERGE
ST1SV	242	52	37628	37922	ADNIMERGE
ST127SV	134	84	37493	37711	ADNIMERGE
LEU	81	51	36427	36559	ADNIMERGE
ST61SV	239	44	35916	36199	ADNIMERGE
BRAIN_STEM_VOLUME	241	51	35612	35904	ADNIMERGE

ST91SA	232	51	35267	35550	ADNIMERGE
ST55SA	242	53	34948	35243	ADNIMERGE
ST59SA	211	47	34914	35172	ADNIMERGE
XL.HDL.PL	124	94	34618	34836	ADNIMERGE
ST46SA	162	42	33933	34137	ADNIMERGE
HDL.C	129	97	32870	33096	ADNIMERGE
ST102SA	143	42	32735	32920	ADNIMERGE
HDL2.C	123	96	32408	32627	ADNIMERGE
C14.1	110	0	32069	32179	ADNIMERGE
LEFT_VENTRALDC_VOLUME	249	47	31558	31854	ADNIMERGE
L.HDL.P	111	98	31198	31407	ADNIMERGE
ST116SA	208	50	31042	31300	ADNIMERGE
L.HDL.L	109	98	30953	31160	ADNIMERGE
XL.HDL.L	130	83	30929	31142	ADNIMERGE
CTX_LH_ISTHMUSCINGULATE_VOLUME	248	47	30823	31118	ADNIMERGE
L.HDL.FC	104	96	30759	30959	ADNIMERGE
ST32SA	232	51	30377	30660	ADNIMERGE
L.HDL.C	103	96	30276	30475	ADNIMERGE
L.HDL.PL	118	98	30089	30305	ADNIMERGE
ST95SA	217	49	29938	30204	ADNIMERGE
L.HDL.CE	103	94	29703	29900	ADNIMERGE
XL.HDL.P	131	77	29483	29691	ADNIMERGE
XL.HDL.FC	132	82	29277	29491	ADNIMERGE
LYSOPC.A.C18.1	147	0	29314	29461	ADNIMERGE
ST50SA	208	28	29216	29452	ADNIMERGE
CREA	7	99	27516	27622	ADNIMERGE
ST21SV	98	61	26656	26815	ADNIMERGE
ST98SA	240	47	25763	26050	ADNIMERGE
PHE	105	0	25889	25994	ADNIMERGE
PE.P.16.0.22.4	127	0	25721	25848	ADNIMERGE
TOTCHO	135	60	25489	25684	ADNIMERGE
HDL.D	115	86	25331	25532	ADNIMERGE
ST107SA	103	25	24770	24898	ADNIMERGE
ST43SA	163	43	24610	24816	ADNIMERGE
GLU	98	0	23624	23722	ADNIMERGE
ST31SA	201	49	23251	23501	ADNIMERGE
CTX_LH_MEDIALORBITOFRONTAL_VOLUME	257	48	22565	22870	ADNIMERGE
PE.18.0.22.4	102	0	21797	21899	ADNIMERGE
ST39CV	340	9	21119	21468	ADNIMERGE

L.HDL.TG	117	49	21118	21284	ADNIMERGE
PC	138	58	20559	20755	ADNIMERGE
LEFT_CHOROID_PLEXUS_VOLUME	75	65	19482	19622	ADNIMERGE
ST76SV	203	54	19351	19608	ADNIMERGE
LEFT_INF_LAT_VENT_VOLUME	215	102	19073	19390	ADNIMERGE
ST57SA	185	44	19074	19303	ADNIMERGE
CTX_LH_INSULA_VOLUME	248	43	18819	19110	ADNIMERGE
CTX_RH_INSULA_VOLUME	262	47	18752	19061	ADNIMERGE
TOTPG	138	60	18432	18630	ADNIMERGE
PRO	22	8	18379	18409	ADNIMERGE
ST82CV	173	38	18119	18330	ADNIMERGE
XL.HDL.C	129	63	17678	17870	ADNIMERGE
ST118SA	216	43	17604	17863	ADNIMERGE
ST97SA	168	29	17274	17471	ADNIMERGE
ST30SV	184	102	16774	17060	ADNIMERGE
RIGHT_INF_LAT_VENT_VOLUME	225	75	16581	16881	ADNIMERGE
PC.AE.C40.5	174	0	16578	16752	ADNIMERGE
ST47SA	59	0	16292	16351	ADNIMERGE
ST36CV	263	42	16015	16320	ADNIMERGE
ST69SV	24	40	15441	15505	ADNIMERGE
ST15SA	175	45	15227	15447	ADNIMERGE
LEFT_LATERAL_VENTRICLE_VOLUME	145	109	15071	15325	ADNIMERGE
ARG	104	0	15210	15314	ADNIMERGE
LYS	105	0	15007	15112	ADNIMERGE
ST91CV	260	40	13931	14231	ADNIMERGE
PE.P.16.0.18.2	129	0	13687	13816	ADNIMERGE
ST26SA	244	44	13311	13599	ADNIMERGE
ST39SA	301	38	13168	13507	ADNIMERGE
RIGHT_THALAMUS_PROPER_VOLUME	256	0	13224	13480	ADNIMERGE
ST99TS	50	0	13381	13431	ADNIMERGE
ST32CV	250	43	12595	12888	ADNIMERGE
ST94CV	233	46	12384	12663	ADNIMERGE
ST89SV	187	124	12099	12410	ADNIMERGE
ST130CV	343	3	11914	12260	ADNIMERGE
ST130SA	304	34	11695	12033	ADNIMERGE
ST114CV	254	48	11678	11980	ADNIMERGE
ST56CV	288	38	11654	11980	ADNIMERGE

ST109TA	137	0	11795	11932	ADNIMERGE
VENTVOL	144	114	11651	11909	ADNIMERGE
GCDCA	92	0	11703	11795	ADNIMERGE
CTX_RH_PRECUNEUS_VOLUME	320	32	11207	11559	ADNIMERGE
ST111CV	324	21	10783	11128	ADNIMERGE
ST37SV	138	117	10730	10985	ADNIMERGE
ST94TA	259	0	10643	10902	ADNIMERGE
PC.AE.C36.3	156	0	10443	10599	ADNIMERGE
PC.AA.C36.1	207	0	10372	10579	ADNIMERGE
VENTRICLES.BL	144	107	9940	10191	ADNIMERGE
VENTRICLES	144	107	9938	10189	ADNIMERGE
ST35TA	233	1	9502	9736	ADNIMERGE
ST34SA	269	25	9141	9435	ADNIMERGE
PC.AE.C38.5	154	0	9138	9292	ADNIMERGE
ST93SA	266	2	8977	9245	ADNIMERGE
PC.AE.C38.4	125	0	8994	9119	ADNIMERGE
ST34CV	320	1	8584	8905	ADNIMERGE
ST95CV	247	32	8424	8703	ADNIMERGE
ST85CV	346	5	8225	8576	ADNIMERGE
CSF_VOLUME	94	54	8200	8348	ADNIMERGE
SM.C24.1	170	2	8151	8323	ADNIMERGE
XL.HDL.CE	129	60	7923	8112	ADNIMERGE
ST129SA	258	46	7756	8060	ADNIMERGE
NON_WM_HYPOINTENSITIES_VOLUME	130	27	7853	8010	ADNIMERGE
ST48SA	116	2	7826	7944	ADNIMERGE
FUSIFORM.BL	317	40	7390	7747	ADNIMERGE
PC.AE.C38.3	170	0	7423	7593	ADNIMERGE
SM..OH..C22.2	176	62	7318	7556	ADNIMERGE
CTX_LH_PRECUNEUS_VOLUME	304	20	7206	7530	ADNIMERGE
LYSOPC.A.C20.4	90	0	7326	7416	ADNIMERGE
DX.BL	251	0	7069	7320	ADNIMERGE
FUSIFORM	313	35	6965	7313	ADNIMERGE
SM.C16.0	171	11	6938	7120	ADNIMERGE
RIGHT_LATERAL_VENTRICLE_VOLUME	128	50	6817	6995	ADNIMERGE
ST17SV	194	47	6611	6852	ADNIMERGE
ST90TS	34	0	6740	6774	ADNIMERGE
ST72SA	172	34	6355	6561	ADNIMERGE
ST62SA	102	5	6314	6421	ADNIMERGE

SM.C16.1	232	62	5922	6216	ADNIMERGE
ST115CV	291	28	5827	6146	ADNIMERGE
ST93CV	303	0	5831	6134	ADNIMERGE
ST96SV	128	71	5885	6084	ADNIMERGE
ST85SA	277	47	5753	6077	ADNIMERGE
ST111TA	243	0	5518	5761	ADNIMERGE
PTDOB	213	616	4930	5759	ADNIMERGE
ST103CV	289	0	5044	5333	ADNIMERGE
ST35CV	238	45	5047	5330	ADNIMERGE
PC.AE.C36.4	146	0	5120	5266	ADNIMERGE
LYSOPC.A.C18.2	84	0	5111	5195	ADNIMERGE
ST54SA	249	19	4832	5100	ADNIMERGE
CTX_RH_LATERALOCCIPITAL_VOLUME	313	25	4754	5092	ADNIMERGE
ST51TA	246	0	4770	5016	ADNIMERGE
ST55CV	278	36	4667	4981	ADNIMERGE
CTX_RH_SUPERIORFRONTAL_VOLUME	312	5	4603	4920	ADNIMERGE
ST103SA	269	3	4629	4901	ADNIMERGE
MIDTEMP	290	37	4573	4900	ADNIMERGE
MIDTEMP.BL	290	37	4572	4899	ADNIMERGE
PC.AE.C36.2	149	0	4733	4882	ADNIMERGE
WM_HYPOINTENSITIES_VOLUME	149	28	4680	4857	ADNIMERGE
ST34TS	116	0	4728	4844	ADNIMERGE
CTX_LH_SUPERIORFRONTAL_VOLUME	312	10	4451	4773	ADNIMERGE
MELGDATE	72	0	4673	4745	ADNIMERGE
LEFT_CEREBELLUM_WHITE_MATTER_VOLUME	216	0	4469	4685	ADNIMERGE
ST23SA	125	41	4507	4673	ADNIMERGE
CTX_LH_ROSTRALANTERIORCINGULATE_VOLUME	212	40	4358	4610	ADNIMERGE
ST15TS	31	0	4563	4594	ADNIMERGE
ST110TS	34	0	4356	4390	ADNIMERGE
CTX_RH_ROSTRALMIDDLEFRONTAL_VOLUME	269	43	4073	4385	ADNIMERGE
ST52CV	294	11	4039	4344	ADNIMERGE
ALA	110	0	4193	4303	ADNIMERGE
CTX_RH_INFERIORPARIETAL_VOLUME	293	27	3962	4282	ADNIMERGE

RIGHT_VENTRALDC_VOLUME	263	40	3957	4260	ADNIMERGE
ST51TS	32	0	4150	4182	ADNIMERGE
ST26CV	314	4	3782	4100	ADNIMERGE
ST55TS	43	0	4032	4075	ADNIMERGE
CTX_LH_ROSTRALMIDDLEFRONTAL_VOLUME	292	18	3687	3997	ADNIMERGE
RIGHT_CEREBELLUM_CORTEX_VOLUME	220	50	3599	3869	ADNIMERGE
VSHEIGHT	48	62	3701	3811	ADNIMERGE
SM.C24.0	148	0	3541	3689	ADNIMERGE
ST54CV	256	0	3425	3681	ADNIMERGE
LONI_STUDY	74	7	3542	3623	ADNIMERGE
SM..OH..C22.1	160	46	3356	3562	ADNIMERGE
ST40CV	266	34	3195	3495	ADNIMERGE
CTX_RH_POSTCENTRAL_VOLUME	288	0	3184	3472	ADNIMERGE
ST108TA	198	0	3245	3443	ADNIMERGE
ST59TA	248	0	3170	3418	ADNIMERGE
CTX_RH_FUSIFORM_VOLUME	302	6	3110	3418	ADNIMERGE
ST101SV	157	23	3226	3406	ADNIMERGE
CTX_RH_MIDDLETEMPORAL_VOLUME	299	9	3077	3385	ADNIMERGE
CTX_LH_MIDDLETEMPORAL_VOLUME	294	2	3015	3311	ADNIMERGE
LONI_IMAGE	74	6	3218	3298	ADNIMERGE
CTX_LH_LATERALOCCIPITAL_VOLUME	302	3	2970	3275	ADNIMERGE
ST99CV	279	33	2959	3271	ADNIMERGE
ST58CV	285	8	2850	3143	ADNIMERGE
CTX_LH_PRECENTRAL_VOLUME	332	0	2761	3093	ADNIMERGE
PC.AE.C34.1	169	0	2884	3053	ADNIMERGE
ST49TA	191	0	2842	3033	ADNIMERGE
CTX_LH_LATERALORBITOFRONTAL_VOLUME	278	28	2723	3029	ADNIMERGE
CTX_RH_PRECENTRAL_VOLUME	323	2	2688	3013	ADNIMERGE
CEREBELLUMGREYMATTER_VOLUME	230	50	2663	2943	ADNIMERGE
CTX_RH_CUNEUS_VOLUME	238	8	2693	2939	ADNIMERGE
ST90CV	296	30	2598	2924	ADNIMERGE

LEFT_THALAMUS_PROPER_VOLUME	259	0	2487	2746	ADNIMERGE
CTX_RH_INFERIORTEMPORAL_VOLUME	276	30	2439	2745	ADNIMERGE
CTX_RH_SUPERIORTEMPORAL_VOLUME	311	0	2419	2730	ADNIMERGE
CTX_RH_ISTHMUSCINGULATE_VOLUME	210	5	2511	2726	ADNIMERGE
LEFT_AMYGDALA_VOLUME	342	0	2363	2705	ADNIMERGE
PC.AE.C34.2	145	2	2556	2703	ADNIMERGE
CTX_LH_POSTCENTRAL_VOLUME	303	0	2359	2662	ADNIMERGE
ST98CV	271	5	2381	2657	ADNIMERGE
TRP	50	0	2535	2585	ADNIMERGE
WHOLECEREBELLUM_VOLUME	258	45	2267	2570	ADNIMERGE
ST38SA	167	0	2391	2558	ADNIMERGE
ST44SA	238	0	2257	2495	ADNIMERGE
HDL3.C	135	54	2287	2476	ADNIMERGE
PC.AE.C38.6	155	0	2278	2433	ADNIMERGE
ST42SV	191	43	2144	2378	ADNIMERGE
ST85TA	279	0	2064	2343	ADNIMERGE
ST50TA	158	0	2167	2325	ADNIMERGE
CTX_LH_FUSIFORM_VOLUME	285	4	2002	2291	ADNIMERGE
CTX_RH_LATERALORBITOFRONTAL_VOLUME	261	27	1931	2219	ADNIMERGE
RIGHT_CEREBELLUM_WHITE_MATTER_VOLUME	209	0	1945	2154	ADNIMERGE
ST13TA	195	0	1910	2105	ADNIMERGE
ST49CV	286	0	1793	2079	ADNIMERGE
CTX_RH_LINGUAL_VOLUME	246	0	1813	2059	ADNIMERGE
CTX_LH_SUPERIORTEMPORAL_VOLUME	313	0	1739	2052	ADNIMERGE
ST59CV	292	2	1731	2025	ADNIMERGE
ST91TS	41	0	1937	1978	ADNIMERGE
ST129CV	308	0	1617	1925	ADNIMERGE
ST108CV	272	0	1619	1891	ADNIMERGE
PC.AE.C34.3	130	5	1739	1874	ADNIMERGE
CTX_LH_POSTERIORCINGULATE_VOLUME	234	0	1619	1853	ADNIMERGE
PC.AA.C38.3	196	34	1622	1852	ADNIMERGE

VENTRICLE_3RD_SUVR	152	2	1695	1849	ADNIMERGE
RIGHT_PALLIDUM_VOLUME	213	43	1559	1815	ADNIMERGE
ST97CV	230	0	1553	1783	ADNIMERGE
ST97TA	198	0	1584	1782	ADNIMERGE
CTX_LH_BANKSSTS_VOLUME	246	0	1527	1773	ADNIMERGE
CTX_RH_SUPERIORPARIETAL_VOLUME	303	0	1460	1763	ADNIMERGE
ST110TA	220	2	1533	1755	ADNIMERGE
M.HDL.FC	70	49	1595	1714	ADNIMERGE
LEFT_CEREBELLUM_CORTEX_VOLUME	218	45	1369	1632	ADNIMERGE
ST58TA	293	1	1337	1631	ADNIMERGE
CTX_LH_SUPRAMARGINAL_VOLUME	291	1	1249	1541	ADNIMERGE
CTX_LH_LINGUAL_VOLUME	254	0	1285	1539	ADNIMERGE
ST117CV	287	2	1223	1512	ADNIMERGE
ST45TS	27	0	1468	1495	ADNIMERGE
ST102TA	161	0	1328	1489	ADNIMERGE
ST32TS	36	0	1372	1408	ADNIMERGE
ST56TS	52	0	1331	1383	ADNIMERGE
SM.C18.1	180	55	1125	1360	ADNIMERGE
ST90TA	240	0	1107	1347	ADNIMERGE
CTX_RH_SUPRAMARGINAL_VOLUME	280	0	1052	1332	ADNIMERGE
ST50CV	225	0	1098	1323	ADNIMERGE
PC.AE.C40.6	143	0	1172	1315	ADNIMERGE
ST113SA	216	0	1095	1311	ADNIMERGE
ST7SV	77	44	1187	1308	ADNIMERGE
CTX_LH_CAUDALMIDDLEFRONTAL_VOLUME	263	0	1011	1274	ADNIMERGE
M.HDL.C	66	40	1155	1261	ADNIMERGE
CTX_LH_INFERIORTEMPORAL_VOLUME	257	43	957	1257	ADNIMERGE
CTX_LH_INFERIORPARIETAL_VOLUME	269	1	978	1248	ADNIMERGE
ST40TS	39	0	1189	1228	ADNIMERGE
MET	48	0	1176	1224	ADNIMERGE
SER	80	0	1133	1213	ADNIMERGE
M.HDL.PL	73	49	1090	1212	ADNIMERGE

CTX_RH_MEDIALORBITOFRONTAL_VOLUME	237	12	956	1205	ADNIMERGE
M.HDL.L	65	45	1083	1193	ADNIMERGE
ST18SV	135	0	1045	1180	ADNIMERGE
ST128SV	118	6	1047	1171	ADNIMERGE
ST14SA	174	2	995	1171	ADNIMERGE
ST51CV	310	0	838	1148	ADNIMERGE
OPTIC_CHIASM_VOLUME	25	0	1121	1146	ADNIMERGE
M.HDL.CE	66	36	988	1090	ADNIMERGE
CTX_LH_SUPERIORPARIETAL_VOLUME	295	0	794	1089	ADNIMERGE
ST26TA	276	0	808	1084	ADNIMERGE
M.HDL.P	66	45	956	1067	ADNIMERGE
ST13SA	141	10	911	1062	ADNIMERGE
ST74TS	31	0	1010	1041	ADNIMERGE
ST110CV	288	1	728	1017	ADNIMERGE
ST116CV	302	0	703	1005	ADNIMERGE
ST52TA	232	0	745	977	ADNIMERGE
CTX_RH_BANKSSTS_VOLUME	227	0	738	965	ADNIMERGE
CSF_SUVR	141	5	764	910	ADNIMERGE
ST74SA	144	5	741	890	ADNIMERGE
ST38CV	224	0	621	845	ADNIMERGE
CTX_RH_PARSOPERCULARIS_VOLUME	223	0	580	803	ADNIMERGE
ST104TS	29	0	747	776	ADNIMERGE
LEFT_PUTAMEN_VOLUME	206	3	563	772	ADNIMERGE
ST119SA	38	2	722	762	ADNIMERGE
ORN	62	0	694	756	ADNIMERGE
ST113CV	211	0	536	747	ADNIMERGE
ST118CV	295	0	447	742	ADNIMERGE
RIGHT_ACCUMBENS_AREA_VOLUME	281	0	448	729	ADNIMERGE
PE.16.0.18.3	156	0	540	696	ADNIMERGE
ST104SA	141	0	545	686	ADNIMERGE
ST77SV	134	0	538	672	ADNIMERGE
PC.AA.C34.3	181	47	421	649	ADNIMERGE
ST31CV	271	0	363	634	ADNIMERGE
CTX_RH_PARACENTRAL_VOLUME	244	0	379	623	ADNIMERGE
RIGHT_AMYGDALA_VOLUME	324	31	255	610	ADNIMERGE

CTX_LH_CUNEUS_VOLUME	214	0	392	606	ADNIMERGE
ST57TA	171	0	431	602	ADNIMERGE
ST103TA	167	0	434	601	ADNIMERGE
ST57CV	286	0	305	591	ADNIMERGE
CTX_RH_CAUDALMIDDLEFRONTAL_VOLUME	229	0	337	566	ADNIMERGE
ST82TA	109	0	438	547	ADNIMERGE
CTX_RH_PARAHIPPOCAMPAL_VOLUME	254	0	279	533	ADNIMERGE
ST44CV	257	0	275	532	ADNIMERGE
CC_MID_ANTERIOR_VOLUME	239	1	280	520	ADNIMERGE
PC.AA.C40.4	153	0	365	518	ADNIMERGE
ST53SV	221	13	282	516	ADNIMERGE
SM.C18.0	165	46	304	515	ADNIMERGE
ST109CV	205	0	307	512	ADNIMERGE
ST45CV	267	0	243	510	ADNIMERGE
ST107CV	148	0	359	507	ADNIMERGE
PC.AE.C40.2	154	0	350	504	ADNIMERGE
CC_CENTRAL_VOLUME	224	1	279	504	ADNIMERGE
CTX_RH_POSTERIORCINGULATE_VOLUME	230	0	261	491	ADNIMERGE
ST118TA	207	0	277	484	ADNIMERGE
ST47TS	29	0	454	483	ADNIMERGE
ST112SV	195	24	262	481	ADNIMERGE
LEFT_PALLIDUM_VOLUME	78	34	366	478	ADNIMERGE
SM	132	44	292	468	ADNIMERGE
ST117TA	278	0	180	458	ADNIMERGE
FREEC	131	30	285	446	ADNIMERGE
ST113TS	34	0	408	442	ADNIMERGE
ST73SA	173	0	256	429	ADNIMERGE
ST45SA	191	0	236	427	ADNIMERGE
ST119TS	22	0	405	427	ADNIMERGE
ST72CV	224	0	195	419	ADNIMERGE
RIGHT_HIPPOCAMPUS_VOLUME	338	0	79	417	ADNIMERGE
CTX_LH_PARSOPERCULARIS_VOLUME	224	0	188	412	ADNIMERGE
ST31TA	218	0	190	408	ADNIMERGE
ST106SA	75	10	319	404	ADNIMERGE
ST38TA	214	0	182	396	ADNIMERGE

PC.AA.C38.5	183	9	196	388	ADNIMERGE
AGE	124	210	46	380	ADNIMERGE
SROI	294	0	86	380	ADNIMERGE
ST91TA	249	0	127	376	ADNIMERGE
CC_MID_POSTERIOR_VOLUME	181	0	193	374	ADNIMERGE
ST99TA	250	0	119	369	ADNIMERGE
ST31TS	33	0	331	364	ADNIMERGE
ST40TA	275	0	88	363	ADNIMERGE
ST12SV	313	0	47	360	ADNIMERGE
PQDATE	52	8	298	358	ADNIMERGE
ST39TS	22	0	336	358	ADNIMERGE
ST118TS	39	0	312	351	ADNIMERGE
ESTC	132	29	189	350	ADNIMERGE
PC.AA.C34.2	48	0	300	348	ADNIMERGE
PC.AA.C40.5	191	8	145	344	ADNIMERGE
CC_ANTERIOR_VOLUME	218	0	125	343	ADNIMERGE
LEFT_HIPPOCAMPUS_VOLUME	300	0	42	342	ADNIMERGE
ST93TS	72	0	260	332	ADNIMERGE
ESTIMATE	317	0	15	332	ADNIMERGE
UPR	317	0	15	332	ADNIMERGE
ST88SV	322	0	7	329	ADNIMERGE
LWR	316	0	13	329	ADNIMERGE
SM..OH..C16.1	140	3	184	327	ADNIMERGE
SERUM.C	131	30	165	326	ADNIMERGE
ST83SA	35	13	277	325	ADNIMERGE
HIPPOCAMPUS	312	0	5	317	ADNIMERGE
HIPPOCAMPUS.BL	307	0	5	312	ADNIMERGE
ST23CV	177	4	128	309	ADNIMERGE
ST98TS	31	0	269	300	ADNIMERGE
ST29SV	296	0	3	299	ADNIMERGE
ST102CV	219	0	79	298	ADNIMERGE
ST13CV	210	0	73	283	ADNIMERGE
ST15CV	225	0	55	280	ADNIMERGE
ST116TA	171	0	106	277	ADNIMERGE
ST56TA	135	0	142	277	ADNIMERGE
MCSUVRWM3	220	0	56	276	ADNIMERGE
TYR	40	0	234	274	ADNIMERGE
CTX_LH_PARACENTRAL_VOLUME	257	0	17	274	ADNIMERGE

CTX_RH_TRANSVERSETEMPORAL_VOLUME	191	0	73	264	ADNIMERGE
LEFT_LATERAL_VENTRICLE_SUVR	126	0	135	261	ADNIMERGE
ST71SV	257	0	3	260	ADNIMERGE
ST72TA	195	0	59	254	ADNIMERGE
CTX_RH_PERICALCARINE_VOLUME	179	0	70	249	ADNIMERGE
MPACCTRAILSB	241	0	0	241	ADNIMERGE
MPACCTRAILSB.BL	241	0	0	241	ADNIMERGE
RIGHT_LATERAL_VENTRICLE_SUVR	125	0	113	238	ADNIMERGE
ST43CV	226	0	9	235	ADNIMERGE
MOCA	231	0	2	233	ADNIMERGE
TOTAL13	227	0	1	228	ADNIMERGE
ADAS13	227	0	1	228	ADNIMERGE
ADAS13.BL	227	0	1	228	ADNIMERGE
ADNI_MEM	224	0	4	228	ADNIMERGE
FDG.BL	220	0	5	225	ADNIMERGE
ST4SV	136	0	86	222	ADNIMERGE
ST83TA	218	0	4	222	ADNIMERGE
RIGHT_PUTAMEN_VOLUME	211	0	10	221	ADNIMERGE
CTX_LH_PARAHIPPOCAMPAL_VOLUME	218	0	1	219	ADNIMERGE
MPACCDIGIT	217	0	0	217	ADNIMERGE
MPACCDIGIT.BL	217	0	0	217	ADNIMERGE
ST107TA	123	0	92	215	ADNIMERGE
USERDATE2	30	183	0	213	ADNIMERGE
ECOGSPMEM	211	0	0	211	ADNIMERGE
HCI_2014	195	0	16	211	ADNIMERGE
ECOGSPTOTAL	210	0	0	210	ADNIMERGE
PQDATTRA	50	8	152	210	ADNIMERGE
CIR	199	7	3	209	ADNIMERGE
CTX_RH_PARSORBITALIS_VOLUME	198	0	10	208	ADNIMERGE
TOTSCORE	206	0	0	206	ADNIMERGE
ADAS11	206	0	0	206	ADNIMERGE
ADAS11.BL	206	0	0	206	ADNIMERGE
ST104CV	190	0	16	206	ADNIMERGE
ST60SA	35	1	169	205	ADNIMERGE
ST24TA	204	0	0	204	ADNIMERGE
ST11SV	203	0	0	203	ADNIMERGE

ST48CV	155	0	48	203	ADNIMERGE
ST117TS	27	0	176	203	ADNIMERGE
ST121CV	180	0	21	201	ADNIMERGE
ST32TA	199	0	1	200	ADNIMERGE
LDELTOTAL	199	0	0	199	ADNIMERGE
LDELTOTAL.BL	199	0	0	199	ADNIMERGE
LEFT_ACCUMBENS_AREA_VOLUME	198	0	0	198	ADNIMERGE
Q4SCORE	198	0	0	198	ADNIMERGE
ADASQ4	198	0	0	198	ADNIMERGE
ADASQ4.BL	198	0	0	198	ADNIMERGE
ENTORHINAL.BL	194	0	1	195	ADNIMERGE
ST15TA	163	0	31	194	ADNIMERGE
ST72TS	46	0	147	193	ADNIMERGE
ENTORHINAL	193	0	0	193	ADNIMERGE
ST70SV	186	0	6	192	ADNIMERGE
CC_POSTERIOR_VOLUME	140	0	51	191	ADNIMERGE
PC.AA.C36.3	170	0	19	189	ADNIMERGE
ECOGSPLANG	189	0	0	189	ADNIMERGE
CTX_LH_ENTORHINAL_VOLUME	188	0	0	188	ADNIMERGE
CTX_LH_PRECUNEUS_SUVR	187	0	0	187	ADNIMERGE
ST74CV	180	0	5	185	ADNIMERGE
CTX_RH_PRECUNEUS_SUVR	184	0	0	184	ADNIMERGE
CTX_LH_BANKSSTS_SUVR	183	0	0	183	ADNIMERGE
CTX_LH_SUPERIORFRONTAL_SUVR	183	0	0	183	ADNIMERGE
CTX_RH_BANKSSTS_SUVR	183	0	0	183	ADNIMERGE
CTX_RH_ROSTRALMIDDLEFRONTAL_SUVR	183	0	0	183	ADNIMERGE
ST83CV	179	0	4	183	ADNIMERGE
CTX_LH_CAUDALMIDDLEFRONTAL_SUVR	181	0	0	181	ADNIMERGE
CTX_LH_ROSTRALMIDDLEFRONTAL_SUVR	181	0	0	181	ADNIMERGE
CTX_RH_SUPERIORFRONTAL_SUVR	181	0	0	181	ADNIMERGE
CTX_RH_CAUDALMIDDLEFRONTAL_SUVR	180	0	0	180	ADNIMERGE
CTX_RH_ENTORHINAL_VOLUME	180	0	0	180	ADNIMERGE

ST121SA	142	0	38	180	ADNIMERGE
ST43TA	154	0	24	178	ADNIMERGE
CTX_RH_MEDIALORBITOFRONTAL_SUVR	178	0	0	178	ADNIMERGE
RIGHT_CHOROID_PLEXUS_SUVR	99	0	79	178	ADNIMERGE
CTX_LH_PARSORBITALIS_VOLUME	178	0	0	178	ADNIMERGE
ECOGSPVISSPAT	177	0	0	177	ADNIMERGE
CTX_LH_INFERIORPARIETAL_SUVR	177	0	0	177	ADNIMERGE
PC.AE.C36.5	137	0	39	176	ADNIMERGE
CTX_LH_INFERIORTEMPORAL_SUVR	176	0	0	176	ADNIMERGE
CTX_RH_INFERIORPARIETAL_SUVR	176	0	0	176	ADNIMERGE
CTX_RH_LATERALORBITOFRONTAL_SUVR	176	0	0	176	ADNIMERGE
SUMMARYSUVR_COMPOSITE_REFNORM	176	0	0	176	ADNIMERGE
CTX_LH_POSTERIORCINGULATE_SUVR	175	0	0	175	ADNIMERGE
ST46TA	84	0	91	175	ADNIMERGE
PUFA	152	20	3	175	ADNIMERGE
ST35TS	43	0	131	174	ADNIMERGE
CTX_LH_LATERALORBITOFRONTAL_SUVR	173	0	0	173	ADNIMERGE
RAVLT.IMMEDIATE	158	12	2	172	ADNIMERGE
RAVLT.IMMEDIATE.BL	158	12	2	172	ADNIMERGE
CTX_RH_SUPERIORPARIETAL_SUVR	172	0	0	172	ADNIMERGE
ST105CV	156	0	15	171	ADNIMERGE
Q13TASKA	151	1	18	170	ADNIMERGE
LIMMTOTAL	170	0	0	170	ADNIMERGE
CTX_RH_INFERIORTEMPORAL_SUVR	170	0	0	170	ADNIMERGE
ST75SV	166	0	4	170	ADNIMERGE
CTX_LH_MEDIALORBITOFRONTAL_SUVR	169	0	0	169	ADNIMERGE
CTX_LH_PERICALCARINE_VOLUME	146	0	23	169	ADNIMERGE
CTX_RH_POSTERIORCINGULATE_SUVR	168	0	0	168	ADNIMERGE
FAQTOTAL	167	0	0	167	ADNIMERGE

FAQ	167	0	0	167	ADNIMERGE
FAQ.BL	167	0	0	167	ADNIMERGE
ST45TA	166	0	0	166	ADNIMERGE
ST24CV	166	0	0	166	ADNIMERGE
ST46CV	165	0	1	166	ADNIMERGE
ST16SV	158	0	7	165	ADNIMERGE
AVTOT6	164	0	0	164	ADNIMERGE
CTX_LH_PARACENTRAL_SUVR	164	0	0	164	ADNIMERGE
CTX_LH_SUPERIORPARIETAL_SUVR	164	0	0	164	ADNIMERGE
AVTOT5	162	1	0	163	ADNIMERGE
CDRSB	162	0	0	162	ADNIMERGE
CDRSB.BL	162	0	0	162	ADNIMERGE
CTX_RH_ISTHMUSCINGULATE_SUVR	162	0	0	162	ADNIMERGE
CTX_RH_PARACENTRAL_SUVR	162	0	0	162	ADNIMERGE
CTX_RH_PARSOPERCULARIS_SUVR	162	0	0	162	ADNIMERGE
CTX_RH_PARSORBITALIS_SUVR	162	0	0	162	ADNIMERGE
CTX_LH_ISTHMUSCINGULATE_SUVR	161	0	0	161	ADNIMERGE
CTX_LH_PARSTRIANGULARIS_SUVR	161	0	0	161	ADNIMERGE
CTX_LH_ROSTRALANTERIORCINGULATE_SUVR	161	0	0	161	ADNIMERGE
CTX_LH_SUPRAMARGINAL_SUVR	161	0	0	161	ADNIMERGE
CTX_RH_ROSTRALANTERIORCINGULATE_SUVR	161	0	0	161	ADNIMERGE
ADNI_EF	160	0	0	160	ADNIMERGE
CTX_LH_MIDDLETEMPORAL_SUVR	160	0	0	160	ADNIMERGE
CTX_LH_PARSOPERCULARIS_SUVR	160	0	0	160	ADNIMERGE
CTX_RH_FUSIFORM_SUVR	160	0	0	160	ADNIMERGE
CTX_LH_PARSTRIANGULARIS_VOLUME	160	0	0	160	ADNIMERGE
TOTFA	152	8	0	160	ADNIMERGE
CTX_LH_PARSORBITALIS_SUVR	159	0	0	159	ADNIMERGE
CTX_RH_LATERALOCCIPITAL_SUVR	159	0	0	159	ADNIMERGE

FAW6	145	10	4	159	ADNIMERGE
CTX_RH_PARSTRIANGULARIS_SUVR	158	0	0	158	ADNIMERGE
CTX_RH_SUPRAMARGINAL_SUVR	158	0	0	158	ADNIMERGE
CTX_LH_FUSIFORM_SUVR	157	0	0	157	ADNIMERGE
MCSUVRCERE	157	0	0	157	ADNIMERGE
VLDL.D	95	46	16	157	ADNIMERGE
ST59TS	39	0	118	157	ADNIMERGE
CTX_LH_SUPERIORTEMPORAL_SUVR	156	0	0	156	ADNIMERGE
CTX_RH_PERICALCARINE_SUVR	155	0	0	155	ADNIMERGE
SFA	149	6	0	155	ADNIMERGE
CTX_LH_INSULA_SUVR	154	0	0	154	ADNIMERGE
CTX_RH_MIDDLETEMPORAL_SUVR	154	0	0	154	ADNIMERGE
CTX_RH_POSTCENTRAL_SUVR	154	0	0	154	ADNIMERGE
CTX_LH_POSTCENTRAL_SUVR	153	0	0	153	ADNIMERGE
CTX_RH_CAUDALANTERIORCINGULATE_SUVR	153	0	0	153	ADNIMERGE
SUMMARYSUVR_WHOLECEREBNORM	153	0	0	153	ADNIMERGE
S.VLDL.CE	140	13	0	153	ADNIMERGE
PC.AA.C34.4	133	18	2	153	ADNIMERGE
CTX_RH_INSULA_SUVR	152	0	0	152	ADNIMERGE
CTX_RH_PRECENTRAL_SUVR	152	0	0	152	ADNIMERGE
CTX_RH_FRONTALPOLE_SUVR	151	0	0	151	ADNIMERGE
CTX_RH_UNKNOWN_VOLUME	151	0	0	151	ADNIMERGE
S.VLDL.C	134	17	0	151	ADNIMERGE
CTX_RH_SUPERIORTEMPORAL_SUVR	150	0	0	150	ADNIMERGE
CTX_LH_PERICALCARINE_SUVR	149	0	0	149	ADNIMERGE
CTX_LH_PRECENTRAL_SUVR	149	0	0	149	ADNIMERGE
CTX_RH_CUNEUS_SUVR	149	0	0	149	ADNIMERGE
TG.PG	89	57	3	149	ADNIMERGE
S.VLDL.FC	123	26	0	149	ADNIMERGE
MUFA	132	17	0	149	ADNIMERGE

CTX_LH_FRONTALPOLE_SUVR	148	0	0	148	ADNIMERGE
M.VLDL.C	118	30	0	148	ADNIMERGE
M.VLDL.CE	128	20	0	148	ADNIMERGE
CTX_LH_LATERALOCCIPITAL_SUVR	147	0	0	147	ADNIMERGE
CTX_RH_LINGUAL_SUVR	147	0	0	147	ADNIMERGE
VLDL.C	129	18	0	147	ADNIMERGE
CTX_LH_CUNEUS_SUVR	146	0	0	146	ADNIMERGE
CTX_RH_TRANSVERSETEMPORAL_SUVR	144	0	0	144	ADNIMERGE
CTX_LH_TRANSVERSETEMPORAL_SUVR	144	0	0	144	ADNIMERGE
S.VLDL.L	114	30	0	144	ADNIMERGE
LA	144	0	0	144	ADNIMERGE
FRONTAL_SUVR	143	0	0	143	ADNIMERGE
LEFT_HIPPOCAMPUS_SUVR	137	0	6	143	ADNIMERGE
CTX_LH_LINGUAL_SUVR	142	0	0	142	ADNIMERGE
S.VLDL.PL	112	30	0	142	ADNIMERGE
Q1SCORE	141	0	0	141	ADNIMERGE
ECOGSPPLAN	141	0	0	141	ADNIMERGE
CTX_LH_CAUDALANTERIORCINGULATE_SUVR	141	0	0	141	ADNIMERGE
PARIETAL_SUVR	140	0	0	140	ADNIMERGE
FAW3	140	0	0	140	ADNIMERGE
ST3SV	100	0	39	139	ADNIMERGE
RIGHT_ACCUMBENS_AREA_SUVR	139	0	0	139	ADNIMERGE
COMPOSITE_SUVR	139	0	0	139	ADNIMERGE
S.VLDL.P	108	31	0	139	ADNIMERGE
M.LDL.TG	135	4	0	139	ADNIMERGE
S.LDL.TG	126	13	0	139	ADNIMERGE
AVTOT4	138	0	0	138	ADNIMERGE
ECOGSPORGAN	138	0	0	138	ADNIMERGE
ACI	138	0	0	138	ADNIMERGE
XXL.VLDL.CE	120	18	0	138	ADNIMERGE
XL.HDL.TG	138	0	0	138	ADNIMERGE
MEAN	136	0	1	137	ADNIMERGE
LEFT_ACCUMBENS_AREA_SUVR	137	0	0	137	ADNIMERGE
TEMPORAL_SUVR	137	0	0	137	ADNIMERGE

PTRTYR	93	0	43	136	ADNIMERGE
CTX_RH_ROSTRALANTERIORCINGULATE_VOLUME	131	0	5	136	ADNIMERGE
RIGHT_HIPPOCAMPUS_SUVR	135	0	1	136	ADNIMERGE
M.VLDL.TG	93	42	0	135	ADNIMERGE
CTX_LH_TRANSVERSETEMPORAL_VOLUME	135	0	0	135	ADNIMERGE
XS.VLDL.P	135	0	0	135	ADNIMERGE
XS.VLDL.L	135	0	0	135	ADNIMERGE
REMNANT.C	135	0	0	135	ADNIMERGE
LEFT_INF_LAT_VENT_SUVR	133	0	1	134	ADNIMERGE
M.VLDL.P	94	40	0	134	ADNIMERGE
L.LDL.TG	133	1	0	134	ADNIMERGE
HDL.TG	120	14	0	134	ADNIMERGE
ST2SV	130	0	3	133	ADNIMERGE
AVDEL30MIN	133	0	0	133	ADNIMERGE
M.VLDL.L	94	39	0	133	ADNIMERGE
M.VLDL.PL	94	39	0	133	ADNIMERGE
APOB.APOA1	116	17	0	133	ADNIMERGE
ST47TA	98	0	34	132	ADNIMERGE
CINGULATE_SUVR	132	0	0	132	ADNIMERGE
XL.VLDL.L	101	31	0	132	ADNIMERGE
APOB	127	5	0	132	ADNIMERGE
XS.VLDL.FC	132	0	0	132	ADNIMERGE
ECOGSPDIVATT	131	0	0	131	ADNIMERGE
ST9SV	27	32	72	131	ADNIMERGE
XXL.VLDL.C	112	19	0	131	ADNIMERGE
LDL.TG	131	0	0	131	ADNIMERGE
ST44TA	103	0	27	130	ADNIMERGE
XL.VLDL.FC	107	23	0	130	ADNIMERGE
IDL.FC	117	0	13	130	ADNIMERGE
TRABSCOR	129	0	0	129	ADNIMERGE
TRABSCOR.BL	129	0	0	129	ADNIMERGE
ST119TA	129	0	0	129	ADNIMERGE
XL.VLDL.P	99	30	0	129	ADNIMERGE
XL.VLDL.PL	103	26	0	129	ADNIMERGE
M.HDL.TG	105	24	0	129	ADNIMERGE
PUFA.FA	97	26	6	129	ADNIMERGE
AVTOT3	122	6	0	128	ADNIMERGE
MEDIAN	127	0	1	128	ADNIMERGE

RIGHT_PUTAMEN_SUVR	128	0	0	128	ADNIMERGE
XL.VLDL.TG	98	30	0	128	ADNIMERGE
L.VLDL.CE	94	34	0	128	ADNIMERGE
L.VLDL.FC	96	32	0	128	ADNIMERGE
FAW6.FA	97	28	3	128	ADNIMERGE
PC.AA.C34.1	128	0	0	128	ADNIMERGE
LA.FA	108	19	1	128	ADNIMERGE
ST55TA	114	0	13	127	ADNIMERGE
S.HDL.TG	90	36	1	127	ADNIMERGE
ST74TA	123	0	4	127	ADNIMERGE
OPTIC_CHIASM_SUVR	123	0	4	127	ADNIMERGE
SERUM.TG	95	32	0	127	ADNIMERGE
XS.VLDL.PL	127	0	0	127	ADNIMERGE
IDL.TG	115	12	0	127	ADNIMERGE
PLA	29	0	97	126	ADNIMERGE
LEFT_PUTAMEN_SUVR	126	0	0	126	ADNIMERGE
XL.VLDL.CE	101	25	0	126	ADNIMERGE
M.VLDL.FC	93	33	0	126	ADNIMERGE
M.LDL.PL	126	0	0	126	ADNIMERGE
CTX_RH_PARAHIPPOCAMPAL_SUVR	126	0	0	126	ADNIMERGE
CTX_LH_PARAHIPPOCAMPAL_SUVR	126	0	0	126	ADNIMERGE
LEFT_CEREBRAL_WHITE_MATTER_SUVR	125	0	0	125	ADNIMERGE
RIGHT_CEREBRAL_WHITE_MATTER_SUVR	125	0	0	125	ADNIMERGE
L.VLDL.C	92	33	0	125	ADNIMERGE
S.VLDL.TG	89	35	1	125	ADNIMERGE
VLDL.TG	90	35	0	125	ADNIMERGE
XS.VLDL.C	125	0	0	125	ADNIMERGE
CTX_LH_UNKNOWN_SUVR	125	0	0	125	ADNIMERGE
ERODED_SUBCORTICALWM_SUVR	124	0	0	124	ADNIMERGE
XXL.VLDL.PL	104	20	0	124	ADNIMERGE
MUFA.FA	91	33	0	124	ADNIMERGE
CTX_RH_UNKNOWN_SUVR	124	0	0	124	ADNIMERGE
LEFT_CAUDATE_SUVR	124	0	0	124	ADNIMERGE
RIGHT_CAUDATE_SUVR	124	0	0	124	ADNIMERGE
ST73TA	24	0	99	123	ADNIMERGE

XS.VLDL.CE	123	0	0	123	ADNIMERGE
RIGHT_INF_LAT_VENT_SUVR	123	0	0	123	ADNIMERGE
L.VLDL.PL	90	33	0	123	ADNIMERGE
S.LDL.PL	123	0	0	123	ADNIMERGE
LEFT_THALAMUS_PROPER_SUVR	123	0	0	123	ADNIMERGE
RIGHT_THALAMUS_PROPER_SUVR	123	0	0	123	ADNIMERGE
ST60TA	122	0	0	122	ADNIMERGE
XL.VLDL.C	98	24	0	122	ADNIMERGE
L.VLDL.P	88	34	0	122	ADNIMERGE
XS.VLDL.TG	94	28	0	122	ADNIMERGE
XXL.VLDL.P	103	18	0	121	ADNIMERGE
XXL.VLDL.L	103	18	0	121	ADNIMERGE
L.VLDL.L	87	34	0	121	ADNIMERGE
L.VLDL.TG	87	34	0	121	ADNIMERGE
RIGHT_PALLIDUM_SUVR	121	0	0	121	ADNIMERGE
LEFT_VESSEL_SUVR	121	0	0	121	ADNIMERGE
DX	120	0	0	120	ADNIMERGE
ST62CV	120	0	0	120	ADNIMERGE
LEFT_CEREBELLUM_CORTEX_SUVR	120	0	0	120	ADNIMERGE
PC.AA.C36.2	76	0	43	119	ADNIMERGE
XXL.VLDL.TG	101	18	0	119	ADNIMERGE
IDL.P	119	0	0	119	ADNIMERGE
RIGHT_CEREBELLUM_CORTEX_SUVR	119	0	0	119	ADNIMERGE
RIGHT_VESSEL_SUVR	119	0	0	119	ADNIMERGE
ST104TA	118	0	0	118	ADNIMERGE
ST129TA	118	0	0	118	ADNIMERGE
CTX_RH_PARSTRIANGULARIS_VOLUME	118	0	0	118	ADNIMERGE
PC.AA.C38.4	91	20	7	118	ADNIMERGE
XXL.VLDL.FC	96	22	0	118	ADNIMERGE
IDL.L	118	0	0	118	ADNIMERGE
LEFT_PALLIDUM_SUVR	118	0	0	118	ADNIMERGE
NON_WM_HYPOINTENSITIES_SUVR	118	0	0	118	ADNIMERGE
RIGHT_VENTRALDC_SUVR	118	0	0	118	ADNIMERGE
ST115TA	105	0	12	117	ADNIMERGE

IDL.CE	117	0	0	117	ADNIMERGE
LEFT_VENTRALDC_SUVR	117	0	0	117	ADNIMERGE
MAX	116	0	0	116	ADNIMERGE
IDL.C	116	0	0	116	ADNIMERGE
L.LDL.P	115	0	0	115	ADNIMERGE
ST93TA	106	0	8	114	ADNIMERGE
CTX_LH_UNKNOWN_VOLUME	114	0	0	114	ADNIMERGE
IDL.PL	114	0	0	114	ADNIMERGE
L.LDL.FC	114	0	0	114	ADNIMERGE
LEFT_AMYGDALA_SUVR	114	0	0	114	ADNIMERGE
CC_CENTRAL_SUVR	113	0	0	113	ADNIMERGE
CTX_LH_TEMPORALPOLE_SUVR	113	0	0	113	ADNIMERGE
CTX_RH_TEMPORALPOLE_SUVR	113	0	0	113	ADNIMERGE
RIGHT_AMYGDALA_SUVR	113	0	0	113	ADNIMERGE
AVTOT2	111	1	0	112	ADNIMERGE
S.LDL.P	112	0	0	112	ADNIMERGE
MMSCORE	111	0	0	111	ADNIMERGE
MMSE	111	0	0	111	ADNIMERGE
MMSE.BL	111	0	0	111	ADNIMERGE
VENTRICLE_4TH_SUVR	111	0	0	111	ADNIMERGE
L.LDL.L	111	0	0	111	ADNIMERGE
L.LDL.PL	111	0	0	111	ADNIMERGE
DHA	111	0	0	111	ADNIMERGE
S.LDL.L	110	0	0	110	ADNIMERGE
L.LDL.CE	110	0	0	110	ADNIMERGE
CC_MID_POSTERIOR_SUVR	110	0	0	110	ADNIMERGE
ST36TA	101	0	8	109	ADNIMERGE
PC.AA.C36.6	105	4	0	109	ADNIMERGE
CC_MID_ANTERIOR_SUVR	109	0	0	109	ADNIMERGE
L.LDL.C	108	0	0	108	ADNIMERGE
ST24SA	104	3	0	107	ADNIMERGE
GP	90	17	0	107	ADNIMERGE
M.LDL.FC	107	0	0	107	ADNIMERGE
CC_ANTERIOR_SUVR	107	0	0	107	ADNIMERGE
ST106CV	105	0	1	106	ADNIMERGE
LEFT_CHOROID_PLEXUS_SUVR	87	0	19	106	ADNIMERGE

M.LDL.P	106	0	0	106	ADNIMERGE
PC.AA.C32.1	105	0	0	105	ADNIMERGE
S.LDL.FC	105	0	0	105	ADNIMERGE
LDL.C	105	0	0	105	ADNIMERGE
COMPOSITE_REF_SUVR	105	0	0	105	ADNIMERGE
M.LDL.L	104	0	0	104	ADNIMERGE
CC_POSTERIOR_SUVR	104	0	0	104	ADNIMERGE
WM_HYPOINTENSITIES_SUVR	104	0	0	104	ADNIMERGE
BRAIN_STEM_SUVR	104	0	0	104	ADNIMERGE
ST5SV	67	0	36	103	ADNIMERGE
ST39TA	102	0	1	103	ADNIMERGE
CTX_LH_ENTORHINAL_SUVR	103	0	0	103	ADNIMERGE
CALLDATE	46	16	39	101	ADNIMERGE
HCI	99	0	2	101	ADNIMERGE
CTX_RH_ENTORHINAL_SUVR	101	0	0	101	ADNIMERGE
ST48TA	81	0	19	100	ADNIMERGE
M.LDL.C	100	0	0	100	ADNIMERGE
S.LDL.C	100	0	0	100	ADNIMERGE
ST130TA	99	0	0	99	ADNIMERGE
LEFT_CEREBELLUM_WHITE_MATTER_SUVR	99	0	0	99	ADNIMERGE
RIGHT_CEREBELLUM_WHITE_MATTER_SUVR	99	0	0	99	ADNIMERGE
ST34TA	97	0	0	97	ADNIMERGE
S.LDL.CE	97	0	0	97	ADNIMERGE
ST14CV	96	0	0	96	ADNIMERGE
Q8SCORE	95	0	0	95	ADNIMERGE
PHS	95	0	0	95	ADNIMERGE
M.LDL.CE	95	0	0	95	ADNIMERGE
CSFVOL	55	0	39	94	ADNIMERGE
FAQFORM	94	0	0	94	ADNIMERGE
ST6SV	73	0	19	92	ADNIMERGE
ST23TA	92	0	0	92	ADNIMERGE
ST68SV	48	0	42	90	ADNIMERGE
UNSAT	82	8	0	90	ADNIMERGE
RAVLT.LEARNING	89	0	0	89	ADNIMERGE
RAVLT.LEARNING.BL	89	0	0	89	ADNIMERGE
ST62TA	88	0	0	88	ADNIMERGE

CDGLOBAL	88	0	0	88	ADNIMERGE
RIGHT_CAUDATE_VOLUME	85	0	3	88	ADNIMERGE
CSF	53	0	34	87	ADNIMERGE
CATANIMSC	87	0	0	87	ADNIMERGE
RAVLT.PERC.FORGETTING	86	0	0	86	ADNIMERGE
RAVLT.PERC.FORGETTING.BL	86	0	0	86	ADNIMERGE
CTX_RH_TEMPORALPOLE_VOLUME	86	0	0	86	ADNIMERGE
ST47CV	84	0	2	86	ADNIMERGE
PC.AA.C36.4	86	0	0	86	ADNIMERGE
CEREBELLUMGREYMATTER_SUVR	84	0	0	84	ADNIMERGE
AVDELTOT	82	0	0	82	ADNIMERGE
LDELCUE	82	0	0	82	ADNIMERGE
CTX_RH_FRONTALPOLE_VOLUME	82	0	0	82	ADNIMERGE
LEFT_CAUDATE_VOLUME	76	0	3	79	ADNIMERGE
ST105TA	78	0	0	78	ADNIMERGE
APOE4	77	0	0	77	ADNIMERGE
ST57TS	32	0	45	77	ADNIMERGE
PRETEMP	1	0	76	77	ADNIMERGE
ST49TS	76	0	0	76	ADNIMERGE
ST95TA	76	0	0	76	ADNIMERGE
ASN	50	0	23	73	ADNIMERGE
ST106TA	70	0	1	71	ADNIMERGE
ST84CV	70	0	1	71	ADNIMERGE
MMFLAGDL	69	0	0	69	ADNIMERGE
ST84SA	64	0	4	68	ADNIMERGE
PE.16.0.22.6	62	1	4	67	ADNIMERGE
PLASMA_NFL	63	2	0	65	ADNIMERGE
PLAVOL	27	0	38	65	ADNIMERGE
BNTSPONT	62	0	0	62	ADNIMERGE
ST121TA	62	0	0	62	ADNIMERGE
CTX_LH_TEMPORALPOLE_VOLUME	62	0	0	62	ADNIMERGE
ST98TA	55	0	6	61	ADNIMERGE
ST108TS	61	0	0	61	ADNIMERGE
PC.AA.C38.6	61	0	0	61	ADNIMERGE
AVTOTB	60	0	0	60	ADNIMERGE
BNTPHON	60	0	0	60	ADNIMERGE
BNTTOTAL	60	0	0	60	ADNIMERGE
MMTREEDL	60	0	0	60	ADNIMERGE

FAQFINAN	60	0	0	60	ADNIMERGE
ST114TA	59	0	0	59	ADNIMERGE
PC.AA.C36.5	59	0	0	59	ADNIMERGE
MMBALLDL	58	0	0	58	ADNIMERGE
CTX_LH_CAUDALANTERIORCINGULATE_VOLUME	57	0	0	57	ADNIMERGE
PE.P.16.0.22.6	57	0	0	57	ADNIMERGE
AVTOT1	56	0	0	56	ADNIMERGE
WHOLECEREBELLUM_SUVR	56	0	0	56	ADNIMERGE
MODE	55	0	0	55	ADNIMERGE
S.HDL.CE	55	0	0	55	ADNIMERGE
NPISCORE	54	0	0	54	ADNIMERGE
ECOGPTMEM	54	0	0	54	ADNIMERGE
ST73CV	54	0	0	54	ADNIMERGE
ST25SA	47	0	7	54	ADNIMERGE
S.HDL.PL	50	4	0	54	ADNIMERGE
PE.P.18.0.20.5	53	0	0	53	ADNIMERGE
ECOGPTTOTAL	53	0	0	53	ADNIMERGE
PE.18.0.20.5	52	0	1	53	ADNIMERGE
NPITOTAL	52	0	0	52	ADNIMERGE
ECOGPTPLAN	51	0	0	51	ADNIMERGE
MIN	50	0	0	50	ADNIMERGE
ST54TS	48	0	1	49	ADNIMERGE
S.HDL.C	49	0	0	49	ADNIMERGE
AVDELERR2	48	0	0	48	ADNIMERGE
ST114TS	48	0	0	48	ADNIMERGE
ST115TS	44	0	0	44	ADNIMERGE
ST52TS	44	0	0	44	ADNIMERGE
CDATE	23	20	0	43	ADNIMERGE
ST36TS	38	0	5	43	ADNIMERGE
ST94TS	29	0	14	43	ADNIMERGE
ST107TS	43	0	0	43	ADNIMERGE
CTX_LH_FRONTALPOLE_VOLUME	42	0	0	42	ADNIMERGE
ST111TS	41	0	0	41	ADNIMERGE
ST60CV	41	0	0	41	ADNIMERGE
KYNURENINE	15	8	18	41	ADNIMERGE
S.HDL.P	40	0	0	40	ADNIMERGE
THR	39	0	0	39	ADNIMERGE
CTX_RH_CAUDALANTERIORCINGULATE_VOLUME	39	0	0	39	ADNIMERGE

ST43TS	15	0	24	39	ADNIMERGE
TRAASCOR	38	0	0	38	ADNIMERGE
BNTCPHON	38	0	0	38	ADNIMERGE
ST119CV	34	0	4	38	ADNIMERGE
ANARTERR	37	0	0	37	ADNIMERGE
ECOGPTVISSPAT	37	0	0	37	ADNIMERGE
ST116TS	28	0	9	37	ADNIMERGE
ST106TS	36	0	0	36	ADNIMERGE
MMDATE	35	0	0	35	ADNIMERGE
ST58TS	32	0	3	35	ADNIMERGE
VENTRICLE_4TH_VOLUME	26	4	5	35	ADNIMERGE
ST73TS	31	0	3	34	ADNIMERGE
AVERRB	34	0	0	34	ADNIMERGE
ST97TS	34	0	0	34	ADNIMERGE
S.HDL.L	34	0	0	34	ADNIMERGE
S.HDL.FC	33	0	0	33	ADNIMERGE
ST129TS	32	0	0	32	ADNIMERGE
ST105TS	22	0	10	32	ADNIMERGE
ST14TS	31	0	0	31	ADNIMERGE
GDTOTAL	30	0	0	30	ADNIMERGE
ORGAN3	30	0	0	30	ADNIMERGE
ECOGPTORGAN	30	0	0	30	ADNIMERGE
ST95TS	30	0	0	30	ADNIMERGE
ST38TS	30	0	0	30	ADNIMERGE
ORGAN2	29	0	0	29	ADNIMERGE
ST48TS	29	0	0	29	ADNIMERGE
ST13TS	17	0	12	29	ADNIMERGE
ST25CV	29	0	0	29	ADNIMERGE
ECOGPTLANG	28	0	0	28	ADNIMERGE
FFLUENCY	28	0	0	28	ADNIMERGE
CLIDATE	25	2	0	27	ADNIMERGE
ECOGPTDIVATT	27	0	0	27	ADNIMERGE
GDMEMORY	26	0	0	26	ADNIMERGE
RNATIME	10	16	0	26	ADNIMERGE
MONDATE	23	2	0	25	ADNIMERGE
POSTTEMP	1	0	24	25	ADNIMERGE
VISSPAT1	23	0	0	23	ADNIMERGE
ST54TA	23	0	0	23	ADNIMERGE
ST50TS	22	0	0	22	ADNIMERGE
FAW3.FA	22	0	0	22	ADNIMERGE
SARCOSINE	13	0	8	21	ADNIMERGE

ALB	20	0	0	20	ADNIMERGE
NPIG	19	0	0	19	ADNIMERGE
VISSPAT2	19	0	0	19	ADNIMERGE
ST85TS	19	0	0	19	ADNIMERGE
ST25TA	19	0	0	19	ADNIMERGE
ST84TA	19	0	0	19	ADNIMERGE
LDL.D	19	0	0	19	ADNIMERGE
RAVLT.FORGETTING	18	0	0	18	ADNIMERGE
RAVLT.FORGETTING.BL	18	0	0	18	ADNIMERGE
BILAVTIME	10	8	0	18	ADNIMERGE
BIREDTIME	10	8	0	18	ADNIMERGE
BILAVCENT	10	8	0	18	ADNIMERGE
BIREDCENT	10	8	0	18	ADNIMERGE
CLTIME	10	8	0	18	ADNIMERGE
ST130TS	17	0	0	17	ADNIMERGE
BILAVFROZ	10	7	0	17	ADNIMERGE
BILAVTRNS	10	7	0	17	ADNIMERGE
BIREDFROZ	10	7	0	17	ADNIMERGE
BIREDTRNS	10	7	0	17	ADNIMERGE
ST83TS	17	0	0	17	ADNIMERGE
MMDAY	15	0	0	15	ADNIMERGE
ST46TS	15	0	0	15	ADNIMERGE
ST26TS	15	0	0	15	ADNIMERGE
DHA.FA	15	0	0	15	ADNIMERGE
FHQPROV	14	0	0	14	ADNIMERGE
NPIC	14	0	0	14	ADNIMERGE
ST24TS	14	0	0	14	ADNIMERGE
ST25TS	13	0	0	13	ADNIMERGE
ST113TA	13	0	0	13	ADNIMERGE
ST14TA	10	0	3	13	ADNIMERGE
ST23TS	13	0	0	13	ADNIMERGE
ST102TS	11	0	0	11	ADNIMERGE
LEFT_VESSEL_VOLUME	4	0	7	11	ADNIMERGE
BICSFTIME	10	0	0	10	ADNIMERGE
ST121TS	10	0	0	10	ADNIMERGE
ST84TS	10	0	0	10	ADNIMERGE
BILAVAMT	9	0	0	9	ADNIMERGE
ST82TS	9	0	0	9	ADNIMERGE
NPII	9	0	0	9	ADNIMERGE
BOHBUT	9	0	0	9	ADNIMERGE

BILAVVOL	8	0	0	8	ADNIMERGE
BNTSTIM	8	0	0	8	ADNIMERGE
SERVOL	8	0	0	8	ADNIMERGE
C2	6	2	0	8	ADNIMERGE
SFA.FA	8	0	0	8	ADNIMERGE
MMFLOOR	7	0	0	7	ADNIMERGE
BIREDAMT	7	0	0	7	ADNIMERGE
ST109TS	7	0	0	7	ADNIMERGE
VBSI	7	0	0	7	ADNIMERGE
GDCA.DCA	7	0	0	7	ADNIMERGE
TDCA.DCA	7	0	0	7	ADNIMERGE
GDCA.CA	7	0	0	7	ADNIMERGE
TDCA.CA	7	0	0	7	ADNIMERGE
GLCA.CDCA	7	0	0	7	ADNIMERGE
TLCA.CDCA	7	0	0	7	ADNIMERGE
MMR	6	0	0	6	ADNIMERGE
NPID	6	0	0	6	ADNIMERGE
SENT_PLA	6	0	0	6	ADNIMERGE
TRABERROM	6	0	0	6	ADNIMERGE
LAC	6	0	0	6	ADNIMERGE
ACE	6	0	0	6	ADNIMERGE
ACACE	6	0	0	6	ADNIMERGE
APVOLUME	5	0	0	5	ADNIMERGE
CLVOLUME	5	0	0	5	ADNIMERGE
TRABERRCOM	5	0	0	5	ADNIMERGE
GLC	5	0	0	5	ADNIMERGE
C18.2	5	0	0	5	ADNIMERGE
STDEV	5	0	0	5	ADNIMERGE
MMO	4	0	0	4	ADNIMERGE
GDHAPPY	4	0	0	4	ADNIMERGE
VSTEMP	4	0	0	4	ADNIMERGE
VSWTUNIT	4	0	0	4	ADNIMERGE
VSTMPUNT	4	0	0	4	ADNIMERGE
POSTDIABP	4	0	0	4	ADNIMERGE
PRESYSTBP	4	0	0	4	ADNIMERGE
POSTSYSTBP	4	0	0	4	ADNIMERGE
RNAVOL	4	0	0	4	ADNIMERGE
LDELBEGIN	4	0	0	4	ADNIMERGE
LIMMEND	4	0	0	4	ADNIMERGE
TIMEEND	4	0	0	4	ADNIMERGE
TIMEBEGAN	4	0	0	4	ADNIMERGE

AVDELBEGAN	4	0	0	4	ADNIMERGE
USED_CSF	4	0	0	4	ADNIMERGE
SENT_CSF	4	0	0	4	ADNIMERGE
BIREDVOL	4	0	0	4	ADNIMERGE
ST103TS	4	0	0	4	ADNIMERGE
ST44TS	4	0	0	4	ADNIMERGE
ST8SV	4	0	0	4	ADNIMERGE
TMCA_A_B	4	0	0	4	ADNIMERGE
DCA.CA	4	0	0	4	ADNIMERGE
MMAREA	3	0	0	3	ADNIMERGE
MMHOSPIT	3	0	0	3	ADNIMERGE
MMREPEAT	3	0	0	3	ADNIMERGE
BCENERGY	3	0	0	3	ADNIMERGE
BCDPMOOD	3	0	0	3	ADNIMERGE
VSBPDIA	3	0	0	3	ADNIMERGE
VSBPSYS	3	0	0	3	ADNIMERGE
PREDIABP	3	0	0	3	ADNIMERGE
ST125SV	3	0	0	3	ADNIMERGE
ST66SV	3	0	0	3	ADNIMERGE
RIGHT_VESSEL_VOLUME	3	0	0	3	ADNIMERGE
NPIH	3	0	0	3	ADNIMERGE
MEASURE_1	3	0	0	3	ADNIMERGE
APGEN1	3	0	0	3	ADNIMERGE
PTEDUCAT	2	0	0	2	ADNIMERGE
GDENERGY	2	0	0	2	ADNIMERGE
DXDEP	2	0	0	2	ADNIMERGE
PREHEART	2	0	0	2	ADNIMERGE
VSPULSE	2	0	0	2	ADNIMERGE
POSTHEART	2	0	0	2	ADNIMERGE
BICSFAMT	2	0	0	2	ADNIMERGE
AVERR6	2	0	0	2	ADNIMERGE
ST62TS	2	0	0	2	ADNIMERGE
BNTCSTIM	2	0	0	2	ADNIMERGE
T4.OH.PRO	2	0	0	2	ADNIMERGE
NPIL	2	0	0	2	ADNIMERGE
CA.CDCA	2	0	0	2	ADNIMERGE
NPIE	2	0	0	2	ADNIMERGE
KMNDBCBBSI	2	0	0	2	ADNIMERGE
HMHYPERT	2	0	0	2	ADNIMERGE
GDAFRAID	1	0	0	1	ADNIMERGE
GDBORED	1	0	0	1	ADNIMERGE

GDDROP	1	0	0	1	ADNIMERGE
GDSATIS	1	0	0	1	ADNIMERGE
GDHOME	1	0	0	1	ADNIMERGE
AVDELERR1	1	0	0	1	ADNIMERGE
AVERR2	1	0	0	1	ADNIMERGE
ST60TS	1	0	0	1	ADNIMERGE
IMAGEUID_LONI	1	0	0	1	ADNIMERGE
STUDYID_LONI	1	0	0	1	ADNIMERGE
LETTERS	1	0	0	1	ADNIMERGE
WELL.POSITION	1	0	0	1	ADNIMERGE
WELL_POSITION	1	0	0	1	ADNIMERGE
C0	1	0	0	1	ADNIMERGE
NPIJ	1	0	0	1	ADNIMERGE
NPIK	1	0	0	1	ADNIMERGE
PMBLGLUC	1	0	0	1	ADNIMERGE
MEASURE_2	1	0	0	1	ADNIMERGE
POSTRESP	1	0	0	1	ADNIMERGE
PRERESP	1	0	0	1	ADNIMERGE
GDSPIRIT	1	0	0	1	ADNIMERGE
MH4CARD	1	0	0	1	ADNIMERGE
BCANKLE	1	0	0	1	ADNIMERGE
PXEDEMA	1	0	0	1	ADNIMERGE
MML	1	0	0	1	ADNIMERGE
MMW	1	0	0	1	ADNIMERGE
MHPSYCH	1	0	0	1	ADNIMERGE
BCMUSCLE	1	0	0	1	ADNIMERGE
MH8MUSCL	1	0	0	1	ADNIMERGE
BCDRYMTH	1	0	0	1	ADNIMERGE

Table S2 – Significant Comparison Frequencies For Features By Maximum Alpha (ADNIMERGE Features Only)

Feature	ADNIMERGE Frequency	Gene Expression Frequency	MRI Frequency	Total Frequency	Domain
L.LDL.L	23	0	0	23	ADNIMERGE
LDL.C	22	0	0	22	ADNIMERGE
L.LDL.P	21	0	0	21	ADNIMERGE
L.LDL.PL	21	0	0	21	ADNIMERGE
L.LDL.C	21	0	0	21	ADNIMERGE
L.LDL.FC	21	0	0	21	ADNIMERGE
S.LDL.P	21	0	0	21	ADNIMERGE
CTX_RH_PARSOPERCULARIS_SUVR	21	0	0	21	ADNIMERGE
M.VLDL.L	20	0	0	20	ADNIMERGE
L.LDL.CE	20	0	0	20	ADNIMERGE
M.LDL.P	20	0	0	20	ADNIMERGE
S.LDL.L	20	0	0	20	ADNIMERGE
CTX_LH_SUPERIORFRONTAL_SUVR	20	0	0	20	ADNIMERGE
CTX_RH_SUPERIORFRONTAL_SUVR	20	0	0	20	ADNIMERGE
CTX_RH_SUPRAMARGINAL_SUVR	20	0	0	20	ADNIMERGE
M.VLDL.FC	19	0	0	19	ADNIMERGE
IDL.PL	19	0	0	19	ADNIMERGE
L.VLDL.P	18	0	0	18	ADNIMERGE
M.VLDL.P	18	0	0	18	ADNIMERGE
VLDL.TG	18	0	0	18	ADNIMERGE
IDL.P	18	0	0	18	ADNIMERGE
M.LDL.L	18	0	0	18	ADNIMERGE
IDL.L	18	0	0	18	ADNIMERGE
CTX_LH_SUPRAMARGINAL_SUVR	18	0	0	18	ADNIMERGE
CTX_LH_POSTERIORCINGULATE_SUVR	18	0	0	18	ADNIMERGE
CTX_LH_CAUDALMIDDLEFRONTAL_SUVR	18	0	0	18	ADNIMERGE
CTX_LH_PARSOPERCULARIS_SUVR	18	0	0	18	ADNIMERGE
L.VLDL.PL	17	0	0	17	ADNIMERGE
L.VLDL.TG	17	0	0	17	ADNIMERGE
M.VLDL.PL	17	0	0	17	ADNIMERGE
S.LDL.C	17	0	0	17	ADNIMERGE
CTX_RH_POSTERIORCINGULATE_SUVR	17	0	0	17	ADNIMERGE
CTX_RH_CAUDALMIDDLEFRONTAL_SUVR	17	0	0	17	ADNIMERGE
CTX_RH_ROSTRALMIDDLEFRONTAL_SUVR	17	0	0	17	ADNIMERGE
XL.VLDL.L	16	0	0	16	ADNIMERGE
L.VLDL.L	16	0	0	16	ADNIMERGE
L.VLDL.FC	16	0	0	16	ADNIMERGE

SERUM.TG	16	0	0	16	ADNIMERGE
M.LDL.C	16	0	0	16	ADNIMERGE
CTX_LH_ROSTRALANTERIORCINGULATE_SUVR	16	0	0	16	ADNIMERGE
CTX_RH_PARSTRIANGULARIS_SUVR	16	0	0	16	ADNIMERGE
CTX_RH_PRECUNEUS_SUVR	16	0	0	16	ADNIMERGE
XL.VLDL.P	15	0	0	15	ADNIMERGE
L.VLDL.C	15	0	0	15	ADNIMERGE
S.VLDL.TG	15	0	0	15	ADNIMERGE
IDL.C	15	0	0	15	ADNIMERGE
IDL.CE	15	0	0	15	ADNIMERGE
M.LDL.CE	14	0	0	14	ADNIMERGE
S.LDL.CE	14	0	0	14	ADNIMERGE
CTX_RH_ROSTRALANTERIORCINGULATE_SUVR	14	0	0	14	ADNIMERGE
CTX_LH_PARSTRIANGULARIS_SUVR	14	0	0	14	ADNIMERGE
XL.VLDL.C	13	0	0	13	ADNIMERGE
M.LDL.FC	13	0	0	13	ADNIMERGE
CTX_LH_PRECUNEUS_SUVR	13	0	0	13	ADNIMERGE
CTX_RH_LATERALORBITOFRONTAL_SUVR	13	0	0	13	ADNIMERGE
M.VLDL.C	12	0	0	12	ADNIMERGE
CTX_LH_ROSTRALMIDDLEFRONTAL_SUVR	12	0	0	12	ADNIMERGE
L.VLDL.CE	11	0	0	11	ADNIMERGE
M.LDL.PL	11	0	0	11	ADNIMERGE
XL.VLDL.PL	10	0	0	10	ADNIMERGE
S.VLDL.P	10	0	0	10	ADNIMERGE
CTX_LH_LATERALORBITOFRONTAL_SUVR	10	0	0	10	ADNIMERGE
XL.VLDL.CE	9	0	0	9	ADNIMERGE
S.VLDL.L	9	0	0	9	ADNIMERGE
IDL.FC	9	0	0	9	ADNIMERGE
FREEC	9	0	0	9	ADNIMERGE
CTX_RH_MEDIALORBITOFRONTAL_SUVR	9	0	0	9	ADNIMERGE
XXL.VLDL.C	8	0	0	8	ADNIMERGE
M.VLDL.TG	8	0	0	8	ADNIMERGE
S.LDL.FC	8	0	0	8	ADNIMERGE
HDL.D	8	0	0	8	ADNIMERGE
L.HDL.L	8	0	0	8	ADNIMERGE
L.HDL.P	8	0	0	8	ADNIMERGE
CTX_RH_BANKSSTS_SUVR	8	0	0	8	ADNIMERGE
CTX_RH_CAUDALANTERIORCINGULATE_SUVR	8	0	0	8	ADNIMERGE
CTX_RH_INFERIORPARIETAL_SUVR	8	0	0	8	ADNIMERGE
CTX_RH_SUPERIORPARIETAL_SUVR	8	0	0	8	ADNIMERGE
CTX_RH_POSTCENTRAL_SUVR	8	0	0	8	ADNIMERGE

EXAMDATE.BL	7	0	0	7	ADNIMERGE
LONISID	7	0	0	7	ADNIMERGE
XXL.VLDL.FC	7	0	0	7	ADNIMERGE
TG.PG	7	0	0	7	ADNIMERGE
CTX_RH_PRECENTRAL_SUVR	7	0	0	7	ADNIMERGE
CTX_RH_ISTHMUSCINGULATE_SUVR	7	0	0	7	ADNIMERGE
PTNO	6	0	0	6	ADNIMERGE
XXL.VLDL.L	6	0	0	6	ADNIMERGE
XXL.VLDL.P	6	0	0	6	ADNIMERGE
XXL.VLDL.PL	6	0	0	6	ADNIMERGE
SERUM.C	6	0	0	6	ADNIMERGE
XL.HDL.L	6	0	0	6	ADNIMERGE
XL.HDL.P	6	0	0	6	ADNIMERGE
L.HDL.C	6	0	0	6	ADNIMERGE
L.HDL.CE	6	0	0	6	ADNIMERGE
CTX_LH_CAUDALANTERIORCINGULATE_SUVR	6	0	0	6	ADNIMERGE
CTX_LH_PRECENTRAL_SUVR	6	0	0	6	ADNIMERGE
CTX_RH_INSULA_SUVR	6	0	0	6	ADNIMERGE
CTX_LH_ISTHMUSCINGULATE_SUVR	6	0	0	6	ADNIMERGE
CTX_LH_PARACENTRAL_SUVR	6	0	0	6	ADNIMERGE
CTX_RH_PARACENTRAL_SUVR	6	0	0	6	ADNIMERGE
CTX_RH_SUPERIORTEMPORAL_SUVR	6	0	0	6	ADNIMERGE
SAMPLE.BAR.CODE	5	0	0	5	ADNIMERGE
SAMPLE.IDENTIFICATION	5	0	0	5	ADNIMERGE
IMAGEUID_1	5	0	0	5	ADNIMERGE
ADAS11	5	0	0	5	ADNIMERGE
TOTSCORE	5	0	0	5	ADNIMERGE
TOTAL13	5	0	0	5	ADNIMERGE
ADAS13	5	0	0	5	ADNIMERGE
ADAS11.BL	5	0	0	5	ADNIMERGE
ADAS13.BL	5	0	0	5	ADNIMERGE
BILAVCENT	5	0	0	5	ADNIMERGE
BIREDCENT	5	0	0	5	ADNIMERGE
BILAVFROZ	5	0	0	5	ADNIMERGE
BILAVTRNS	5	0	0	5	ADNIMERGE
BIREDFROZ	5	0	0	5	ADNIMERGE
BIREDTRNS	5	0	0	5	ADNIMERGE
XXL.VLDL.TG	5	0	0	5	ADNIMERGE
XL.VLDL.FC	5	0	0	5	ADNIMERGE
XL.VLDL.TG	5	0	0	5	ADNIMERGE
ESTC	5	0	0	5	ADNIMERGE

XL.HDL.FC	5	0	0	5	ADNIMERGE
L.HDL.FC	5	0	0	5	ADNIMERGE
M.HDL.L	5	0	0	5	ADNIMERGE
M.HDL.P	5	0	0	5	ADNIMERGE
M.HDL.C	5	0	0	5	ADNIMERGE
M.HDL.FC	5	0	0	5	ADNIMERGE
CC_MID_ANTERIOR_SUVR	5	0	0	5	ADNIMERGE
CC_POSTERIOR_SUVR	5	0	0	5	ADNIMERGE
CTX_LH_SUPERIORPARIETAL_SUVR	5	0	0	5	ADNIMERGE
CTX_RH_MIDDLETEMPORAL_SUVR	5	0	0	5	ADNIMERGE
CTX_LH_MEDIALORBITOFRONTAL_SUVR	5	0	0	5	ADNIMERGE
CTX_LH_POSTCENTRAL_SUVR	5	0	0	5	ADNIMERGE
FRONTAL_SUVR	5	0	0	5	ADNIMERGE
COMPOSITE_SUVR	5	0	0	5	ADNIMERGE
SAMPLE_BAR_CODE	4	0	0	4	ADNIMERGE
VENTVOL	4	0	0	4	ADNIMERGE
VENTRICLES	4	0	0	4	ADNIMERGE
VENTRICLES.BL	4	0	0	4	ADNIMERGE
S.LDL.PL	4	0	0	4	ADNIMERGE
XL.HDL.PL	4	0	0	4	ADNIMERGE
XL.HDL.C	4	0	0	4	ADNIMERGE
XL.HDL.CE	4	0	0	4	ADNIMERGE
L.HDL.PL	4	0	0	4	ADNIMERGE
M.HDL.PL	4	0	0	4	ADNIMERGE
M.HDL.CE	4	0	0	4	ADNIMERGE
CC_ANTERIOR_SUVR	4	0	0	4	ADNIMERGE
CC_CENTRAL_SUVR	4	0	0	4	ADNIMERGE
CTX_LH_INFERIORTEMPORAL_SUVR	4	0	0	4	ADNIMERGE
CTX_LH_INFERIORPARIETAL_SUVR	4	0	0	4	ADNIMERGE
CTX_RH_INFERIORTEMPORAL_SUVR	4	0	0	4	ADNIMERGE
CTX_RH_PARSORBITALIS_SUVR	4	0	0	4	ADNIMERGE
BRAIN_STEM_SUVR	4	0	0	4	ADNIMERGE
CINGULATE_SUVR	4	0	0	4	ADNIMERGE
PARIETAL_SUVR	4	0	0	4	ADNIMERGE
TEMPORAL_SUVR	4	0	0	4	ADNIMERGE
SUMMARYSUVR_WHOLECEREBNORM	4	0	0	4	ADNIMERGE
IMAGEUID_2	3	0	0	3	ADNIMERGE
MPACCDIGIT	3	0	0	3	ADNIMERGE
MPACCTRAILSB	3	0	0	3	ADNIMERGE
MPACCDIGIT.BL	3	0	0	3	ADNIMERGE
MPACCTRAILSB.BL	3	0	0	3	ADNIMERGE

RAVLT.IMMEDIATE	3	0	0	3	ADNIMERGE
RAVLT.IMMEDIATE.BL	3	0	0	3	ADNIMERGE
HIPPOCAMPUS	3	0	0	3	ADNIMERGE
HIPPOCAMPUS.BL	3	0	0	3	ADNIMERGE
ST37SV	3	0	0	3	ADNIMERGE
ST96SV	3	0	0	3	ADNIMERGE
REMNANT.C	3	0	0	3	ADNIMERGE
VLDL.D	3	0	0	3	ADNIMERGE
VLDL.C	3	0	0	3	ADNIMERGE
S.VLDL.FC	3	0	0	3	ADNIMERGE
XS.VLDL.L	3	0	0	3	ADNIMERGE
LDL.TG	3	0	0	3	ADNIMERGE
HDL.C	3	0	0	3	ADNIMERGE
HDL2.C	3	0	0	3	ADNIMERGE
WM_HYPOINTENSITIES_SUVR	3	0	0	3	ADNIMERGE
CC_MID_POSTERIOR_SUVR	3	0	0	3	ADNIMERGE
CTX_LH_MIDDLETEMPORAL_SUVR	3	0	0	3	ADNIMERGE
CTX_LH_INSULA_SUVR	3	0	0	3	ADNIMERGE
CEREBELLUMGREYMATTER_VOLUME	3	0	0	3	ADNIMERGE
MMSCORE	2	0	0	2	ADNIMERGE
MMSE	2	0	0	2	ADNIMERGE
MMSE.BL	2	0	0	2	ADNIMERGE
AVTOT3	2	0	0	2	ADNIMERGE
AVTOT4	2	0	0	2	ADNIMERGE
AVDEL30MIN	2	0	0	2	ADNIMERGE
RAVLT.PERC.FORGETTING	2	0	0	2	ADNIMERGE
RAVLT.PERC.FORGETTING.BL	2	0	0	2	ADNIMERGE
ADASQ4	2	0	0	2	ADNIMERGE
Q4SCORE	2	0	0	2	ADNIMERGE
ADASQ4.BL	2	0	0	2	ADNIMERGE
FAQ	2	0	0	2	ADNIMERGE
FAQTOTAL	2	0	0	2	ADNIMERGE
FAQ.BL	2	0	0	2	ADNIMERGE
BILAVTIME	2	0	0	2	ADNIMERGE
BIREDTIME	2	0	0	2	ADNIMERGE
CLTIME	2	0	0	2	ADNIMERGE
IMAGEUID	2	0	0	2	ADNIMERGE
IMAGEUID.BL	2	0	0	2	ADNIMERGE
ICV	2	0	0	2	ADNIMERGE
ST10CV	2	0	0	2	ADNIMERGE
ICV.BL	2	0	0	2	ADNIMERGE

ST29SV	2	0	0	2	ADNIMERGE
ST88SV	2	0	0	2	ADNIMERGE
ESTIMATE	2	0	0	2	ADNIMERGE
LWR	2	0	0	2	ADNIMERGE
UPR	2	0	0	2	ADNIMERGE
BRAINVOL	2	0	0	2	ADNIMERGE
WHOLEBRAIN	2	0	0	2	ADNIMERGE
WHOLEBRAIN.BL	2	0	0	2	ADNIMERGE
BNTPHON	2	0	0	2	ADNIMERGE
BNTSPONT	2	0	0	2	ADNIMERGE
BNTTOTAL	2	0	0	2	ADNIMERGE
XXL.VLDL.CE	2	0	0	2	ADNIMERGE
M.VLDL.CE	2	0	0	2	ADNIMERGE
S.VLDL.C	2	0	0	2	ADNIMERGE
XS.VLDL.P	2	0	0	2	ADNIMERGE
XS.VLDL.PL	2	0	0	2	ADNIMERGE
L.LDL.TG	2	0	0	2	ADNIMERGE
M.LDL.TG	2	0	0	2	ADNIMERGE
PC	2	0	0	2	ADNIMERGE
TOTPG	2	0	0	2	ADNIMERGE
TOTCHO	2	0	0	2	ADNIMERGE
PUFA	2	0	0	2	ADNIMERGE
TOTFA	2	0	0	2	ADNIMERGE
FAW6	2	0	0	2	ADNIMERGE
MEAN	2	0	0	2	ADNIMERGE
MEDIAN	2	0	0	2	ADNIMERGE
MAX	2	0	0	2	ADNIMERGE
ERODED_SUBCORTICALWM_SUVR	2	0	0	2	ADNIMERGE
LEFT_CEREBRAL_WHITE_MATTER_SUVR	2	0	0	2	ADNIMERGE
RIGHT_CEREBRAL_WHITE_MATTER_SUVR	2	0	0	2	ADNIMERGE
CTX_LH_BANKSSTS_SUVR	2	0	0	2	ADNIMERGE
CTX_LH_FUSIFORM_SUVR	2	0	0	2	ADNIMERGE
CTX_RH_FUSIFORM_SUVR	2	0	0	2	ADNIMERGE
CTX_LH_SUPERIORTEMPORAL_SUVR	2	0	0	2	ADNIMERGE
LEFT_CEREBELLUM_CORTEX_VOLUME	2	0	0	2	ADNIMERGE
RIGHT_CEREBELLUM_CORTEX_VOLUME	2	0	0	2	ADNIMERGE
LEFT_CEREBELLUM_WHITE_MATTER_SUVR	2	0	0	2	ADNIMERGE
RIGHT_CEREBELLUM_WHITE_MATTER_SUVR	2	0	0	2	ADNIMERGE
LEFT_VENTRALDC_SUVR	2	0	0	2	ADNIMERGE
RIGHT_VENTRALDC_SUVR	2	0	0	2	ADNIMERGE
AGE	1	0	0	1	ADNIMERGE

PTDOB	1	0	0	1	ADNIMERGE
LDELTOTAL	1	0	0	1	ADNIMERGE
LDELTOTAL.BL	1	0	0	1	ADNIMERGE
CDRSB	1	0	0	1	ADNIMERGE
CDRSB.BL	1	0	0	1	ADNIMERGE
LDELBEGIN	1	0	0	1	ADNIMERGE
LIMMEND	1	0	0	1	ADNIMERGE
CSF	1	0	0	1	ADNIMERGE
CSFVOL	1	0	0	1	ADNIMERGE
RAVLT.LEARNING	1	0	0	1	ADNIMERGE
RAVLT.LEARNING.BL	1	0	0	1	ADNIMERGE
RAVLT.FORGETTING	1	0	0	1	ADNIMERGE
RAVLT.FORGETTING.BL	1	0	0	1	ADNIMERGE
TRABSCOR	1	0	0	1	ADNIMERGE
TRABSCOR.BL	1	0	0	1	ADNIMERGE
ST127SV	1	0	0	1	ADNIMERGE
VENTRICLE_3RD_VOLUME	1	0	0	1	ADNIMERGE
ST17SV	1	0	0	1	ADNIMERGE
ST76SV	1	0	0	1	ADNIMERGE
ST18SV	1	0	0	1	ADNIMERGE
ST77SV	1	0	0	1	ADNIMERGE
BRAIN_STEM_VOLUME	1	0	0	1	ADNIMERGE
ST1SV	1	0	0	1	ADNIMERGE
ST9SV	1	0	0	1	ADNIMERGE
VENTRICLE_4TH_VOLUME	1	0	0	1	ADNIMERGE
PLA	1	0	0	1	ADNIMERGE
PLAVOL	1	0	0	1	ADNIMERGE
URN	1	0	0	1	ADNIMERGE
URNVOL	1	0	0	1	ADNIMERGE
LONI_IMAGE	1	0	0	1	ADNIMERGE
LONI_STUDY	1	0	0	1	ADNIMERGE
IMAGEUID_LONI	1	0	0	1	ADNIMERGE
STUDYID_LONI	1	0	0	1	ADNIMERGE
ECOGSPMEM	1	0	0	1	ADNIMERGE
ECOGSPTOTAL	1	0	0	1	ADNIMERGE
WELL.POSITION	1	0	0	1	ADNIMERGE
WELL_POSITION	1	0	0	1	ADNIMERGE
PC.AE.C34.2	1	0	0	1	ADNIMERGE
PC.AE.C36.3	1	0	0	1	ADNIMERGE
PC.AE.C36.4	1	0	0	1	ADNIMERGE
PC.AE.C38.5	1	0	0	1	ADNIMERGE

C13.PE.16.0.22.6	1	0	0	1	ADNIMERGE
C13.PE.P.16.0.22.6	1	0	0	1	ADNIMERGE
APOB	1	0	0	1	ADNIMERGE
GDCA.CA	1	0	0	1	ADNIMERGE
TDCA.CA	1	0	0	1	ADNIMERGE
S.VLDL.PL	1	0	0	1	ADNIMERGE
S.VLDL.CE	1	0	0	1	ADNIMERGE
XS.VLDL.C	1	0	0	1	ADNIMERGE
XS.VLDL.CE	1	0	0	1	ADNIMERGE
XS.VLDL.TG	1	0	0	1	ADNIMERGE
IDL.TG	1	0	0	1	ADNIMERGE
S.HDL.L	1	0	0	1	ADNIMERGE
S.HDL.P	1	0	0	1	ADNIMERGE
S.HDL.C	1	0	0	1	ADNIMERGE
S.HDL.CE	1	0	0	1	ADNIMERGE
SFA	1	0	0	1	ADNIMERGE
DHA	1	0	0	1	ADNIMERGE
FAW3	1	0	0	1	ADNIMERGE
LA	1	0	0	1	ADNIMERGE
ENTORHINAL	1	0	0	1	ADNIMERGE
ENTORHINAL.BL	1	0	0	1	ADNIMERGE
FUSIFORM	1	0	0	1	ADNIMERGE
FUSIFORM.BL	1	0	0	1	ADNIMERGE
MIDTEMP	1	0	0	1	ADNIMERGE
MIDTEMP.BL	1	0	0	1	ADNIMERGE
MIN	1	0	0	1	ADNIMERGE
MODE	1	0	0	1	ADNIMERGE
CTX_LH_CUNEUS_SUVR	1	0	0	1	ADNIMERGE
CTX_RH_CUNEUS_SUVR	1	0	0	1	ADNIMERGE
CTX_LH_PARSORBITALIS_SUVR	1	0	0	1	ADNIMERGE
CTX_LH_PERICALCARINE_SUVR	1	0	0	1	ADNIMERGE
CTX_RH_PERICALCARINE_SUVR	1	0	0	1	ADNIMERGE
CTX_LH_TRANSVERSETEMPORAL_SUVR	1	0	0	1	ADNIMERGE
CTX_RH_TRANSVERSETEMPORAL_SUVR	1	0	0	1	ADNIMERGE
CTX_LH_FRONTALPOLE_SUVR	1	0	0	1	ADNIMERGE
CTX_RH_FRONTALPOLE_SUVR	1	0	0	1	ADNIMERGE
LEFT_ACCUMBENS_AREA_SUVR	1	0	0	1	ADNIMERGE
RIGHT_ACCUMBENS_AREA_SUVR	1	0	0	1	ADNIMERGE
LEFT_CEREBELLUM_WHITE_MATTER_VOLUME	1	0	0	1	ADNIMERGE
RIGHT_CEREBELLUM_WHITE_MATTER_VOLUME	1	0	0	1	ADNIMERGE
LEFT_CEREBRAL_WHITE_MATTER_VOLUME	1	0	0	1	ADNIMERGE

RIGHT_CEREBRAL_WHITE_MATTER_VOLUME	1	0	0	1	ADNIMERGE
LEFT_HIPPOCAMPUS_SUVR	1	0	0	1	ADNIMERGE
RIGHT_HIPPOCAMPUS_SUVR	1	0	0	1	ADNIMERGE
LEFT_LATERAL_VENTRICLE_SUVR	1	0	0	1	ADNIMERGE
RIGHT_LATERAL_VENTRICLE_SUVR	1	0	0	1	ADNIMERGE
LEFT_PUTAMEN_SUVR	1	0	0	1	ADNIMERGE
RIGHT_PUTAMEN_SUVR	1	0	0	1	ADNIMERGE
WHOLECEREBELLUM_VOLUME	1	0	0	1	ADNIMERGE
MCSUVRCERE	1	0	0	1	ADNIMERGE
ACI	1	0	0	1	ADNIMERGE
PQDATE	1	0	0	1	ADNIMERGE
PQDATTRA	1	0	0	1	ADNIMERGE
TIMEBEGAN	1	0	0	1	ADNIMERGE
TIMEEND	1	0	0	1	ADNIMERGE

Table S3: Comparison Frequencies Of The Subset Analysis (Male Subset)

Domain	Average	Standard Deviation	Minimum	Maximum
ADNIMERGE	1.42	0.63	1.0	4.0
Gene Expression	0.0	0.0	0.0	0.0
MRI	0.0	0.0	0.0	0.0

A – ADNIMERGE Frequencies

Domain	Average	Standard Deviation	Minimum	Maximum
ADNIMERGE	0.0	0.0	0.0	0.0
Gene Expression	1.59	1.13	1.0	6.0
MRI	0.0	0.0	0.0	0.0

B – Gene Expression Frequencies

Domain	Average	Standard Deviation	Minimum	Maximum
ADNIMERGE	0.0	0.0	0.0	0.0
Gene Expression	0.0	0.0	0.0	0.0
MRI	3327.39	2214.28	1.0	4945.0

C – MRI Frequencies

Domain	Average	Standard Deviation	Minimum	Maximum
ADNIMERGE	1.42	0.63	1.0	4.0
Gene Expression	1.59	1.13	1.0	6.0
MRI	3327.39	2214.28	1.0	4945.0

D – Total Frequencies

Table S3 – Male Subset

Table S4: Comparison Frequencies Of The Subset Analysis (Female Subset)

Domain	Average	Standard Deviation	Minimum	Maximum
ADNIMERGE	1.28	0.47	1.0	3.0
Gene Expression	0.0	0.0	0.0	0.0
MRI	0.0	0.0	0.0	0.0

A – ADNIMERGE Frequencies

Domain	Average	Standard Deviation	Minimum	Maximum
ADNIMERGE	0.0	0.0	0.0	0.0
Gene Expression	1.33	0.55	1.0	3.0
MRI	0.0	0.0	0.0	0.0

B – Gene Expression Frequencies

Domain	Average	Standard Deviation	Minimum	Maximum
ADNIMERGE	0.0	0.0	0.0	0.0
Gene Expression	0.0	0.0	0.0	0.0
MRI	4050.25	1929.62	1.0	5146.0

C – MRI Frequencies

Domain	Average	Standard Deviation	Minimum	Maximum
ADNIMERGE	1.28	0.47	1.0	3.0
Gene Expression	1.33	0.55	1.0	3.0
MRI	4050.25	1929.62	1.0	5146.0

D – Total Frequencies

Table S4 – Female Subset

Table S5: Comparison Frequencies Of The Subset Analysis (CDR=0 Subset)

Domain	Average	Standard Deviation	Minimum	Maximum
ADNIMERGE	5.48	5.83	1.0	23.0
Gene Expression	0.0	0.0	0.0	0.0
MRI	0.0	0.0	0.0	0.0

A – ADNIMERGE Frequencies

Domain	Average	Standard Deviation	Minimum	Maximum
ADNIMERGE	0.0	0.0	0.0	0.0
Gene Expression	1.55	0.94	1.0	7.0
MRI	0.0	0.0	0.0	0.0

B – Gene Expression Frequencies

Domain	Average	Standard Deviation	Minimum	Maximum
ADNIMERGE	0.0	0.0	0.0	0.0
Gene Expression	0.0	0.0	0.0	0.0
MRI	2457.08	4397.99	1.0	11957.0

C – MRI Frequencies

Domain	Average	Standard Deviation	Minimum	Maximum
ADNIMERGE	5.48	5.83	1.0	23.0
Gene Expression	1.55	0.94	1.0	7.0
MRI	2457.08	4397.99	1.0	11957.0

D – Total Frequencies

Table S5 – CDR=0 Control Subset

Table S6: Comparison Frequencies Of The Subset Analysis (CDR=0.5 Subset)

Domain	Average	Standard Deviation	Minimum	Maximum
ADNIMERGE	5.48	5.83	1.0	23.0
Gene Expression	0.0	0.0	0.0	0.0
MRI	0.0	0.0	0.0	0.0

A – ADNIMERGE Frequencies

Domain	Average	Standard Deviation	Minimum	Maximum
ADNIMERGE	0.0	0.0	0.0	0.0
Gene Expression	1.55	0.94	1.0	7.0
MRI	0.0	0.0	0.0	0.0

B – Gene Expression Frequencies

Domain	Average	Standard Deviation	Minimum	Maximum
ADNIMERGE	0.0	0.0	0.0	0.0
Gene Expression	0.0	0.0	0.0	0.0
MRI	2457.08	4397.99	1.0	11957.0

C – MRI Frequencies

Domain	Average	Standard Deviation	Minimum	Maximum
ADNIMERGE	5.48	5.83	1.0	23.0
Gene Expression	1.55	0.94	1.0	7.0
MRI	2457.08	4397.99	1.0	11957.0

D – MRI Frequency

Table S6 – CDR=0.5 Subset

Table S7: Comparison Frequencies Of The Subset Analysis (CDR≥1.0 Subset)

Domain	Average	Standard Deviation	Minimum	Maximum
ADNIMERGE	1.67	0.47	1.0	2.0
Gene Expression	0.0	0.0	0.0	0.0
MRI	0.0	0.0	0.0	0.0

A – ADNIMERGE Frequencies

Domain	Average	Standard Deviation	Minimum	Maximum
ADNIMERGE	0.0	0.0	0.0	0.0
Gene Expression	0.0	0.0	0.0	0.0
MRI	0.0	0.0	0.0	0.0

B – Gene Expression Frequencies

Domain	Average	Standard Deviation	Minimum	Maximum
ADNIMERGE	0.0	0.0	0.0	0.0
Gene Expression	0.0	0.0	0.0	0.0
MRI	1.65	1.03	1.0	4.0

C – MRI Frequencies

Domain	Average	Standard Deviation	Minimum	Maximum
ADNIMERGE	1.67	0.47	1.0	2.0
Gene Expression	0.0	0.0	0.0	0.0
MRI	1.65	1.03	1.0	4.0

D – Total Frequencies

Table S7 – CDR≥1.0 Subset

Table S8– Maximally-significant correlations with sufficient data to perform subsetting: Males

Domain	Average	Standard Deviation	Minimum	Maximum
ADNIMERGE	5.48	5.83	1.0	23.0
Gene Expression	0.0	0.0	0.0	0.0
MRI	0.0	0.0	0.0	0.0

A – ADNIMERGE

Domain	Average	Standard Deviation	Minimum	Maximum
ADNIMERGE	0.0	0.0	0.0	0.0
Gene Expression	1.55	0.94	1.0	7.0
MRI	0.0	0.0	0.0	0.0

B – Gene Expression

Domain	Average	Standard Deviation	Minimum	Maximum
ADNIMERGE	0.0	0.0	0.0	0.0
Gene Expression	0.0	0.0	0.0	0.0
MRI	2457.08	4397.99	1.0	11957.0

C – MRI

Domain	Average	Standard Deviation	Minimum	Maximum
ADNIMERGE	5.48	5.83	1.0	23.0
Gene Expression	1.55	0.94	1.0	7.0
MRI	2457.08	4397.99	1.0	11957.0

D - Total

Table S8 – Male Subset

Table S9– Maximally-significant correlations with sufficient data to perform subsetting: Females

Domain	Average	Standard Deviation	Minimum	Maximum
ADNIMERGE	5.48	5.83	1.0	23.0
Gene Expression	0.0	0.0	0.0	0.0
MRI	0.0	0.0	0.0	0.0

A – ADNIMERGE

Domain	Average	Standard Deviation	Minimum	Maximum
ADNIMERGE	0.0	0.0	0.0	0.0
Gene Expression	1.55	0.94	1.0	7.0
MRI	0.0	0.0	0.0	0.0

B – Gene Expression

Domain	Average	Standard Deviation	Minimum	Maximum
ADNIMERGE	0.0	0.0	0.0	0.0
Gene Expression	0.0	0.0	0.0	0.0
MRI	2457.08	4397.99	1.0	11957.0

C – MRI

Domain	Average	Standard Deviation	Minimum	Maximum
ADNIMERGE	5.48	5.83	1.0	23.0
Gene Expression	1.55	0.94	1.0	7.0
MRI	2457.08	4397.99	1.0	11957.0

D - Total

Table S9 – Female Subset

Table S10– Maximally-significant correlations with sufficient data to perform subsetting: CDR=0

Domain	Average	Standard Deviation	Minimum	Maximum
ADNIMERGE	5.48	5.83	1.0	23.0
Gene Expression	0.0	0.0	0.0	0.0
MRI	0.0	0.0	0.0	0.0

A - ADNIMERGE

Domain	Average	Standard Deviation	Minimum	Maximum
ADNIMERGE	0.0	0.0	0.0	0.0
Gene Expression	1.55	0.94	1.0	7.0
MRI	0.0	0.0	0.0	0.0

B – Gene Expression

Domain	Average	Standard Deviation	Minimum	Maximum
ADNIMERGE	0.0	0.0	0.0	0.0
Gene Expression	0.0	0.0	0.0	0.0
MRI	2457.08	4397.99	1.0	11957.0

C – MRI

Domain	Average	Standard Deviation	Minimum	Maximum
ADNIMERGE	5.48	5.83	1.0	23.0
Gene Expression	1.55	0.94	1.0	7.0
MRI	2457.08	4397.99	1.0	11957.0

D - Total

Table S10 – CDR=0 Subset

Table S11– Maximally-significant correlations with sufficient data to perform subsetting: CDR=0.5

Domain	Average	Standard Deviation	Minimum	Maximum
ADNIMERGE	5.48	5.83	1.0	23.0
Gene Expression	0.0	0.0	0.0	0.0
MRI	0.0	0.0	0.0	0.0

A – ADNIMERGE

Domain	Average	Standard Deviation	Minimum	Maximum
ADNIMERGE	0.0	0.0	0.0	0.0
Gene Expression	1.55	0.94	1.0	7.0
MRI	0.0	0.0	0.0	0.0

B – Gene Expression

Domain	Average	Standard Deviation	Minimum	Maximum
ADNIMERGE	0.0	0.0	0.0	0.0
Gene Expression	0.0	0.0	0.0	0.0
MRI	2457.08	4397.99	1.0	11957.0

C – MRI

Domain	Average	Standard Deviation	Minimum	Maximum
ADNIMERGE	5.48	5.83	1.0	23.0
Gene Expression	1.55	0.94	1.0	7.0
MRI	2457.08	4397.99	1.0	11957.0

D - Total

Table S11 – CDR=0.5 Subset

Table S12– Maximally-significant correlations with sufficient data to perform subsetting: CDR≥1.0

Domain	Average	Standard Deviation	Minimum	Maximum
ADNIMERGE	5.51	5.83	1.0	23.0
Gene Expression	0.0	0.0	0.0	0.0
MRI	0.0	0.0	0.0	0.0

A – ADNIMERGE

Domain	Average	Standard Deviation	Minimum	Maximum
ADNIMERGE	0.0	0.0	0.0	0.0
Gene Expression	1.55	0.94	1.0	7.0
MRI	0.0	0.0	0.0	0.0

B – Gene Expression

Domain	Average	Standard Deviation	Minimum	Maximum
ADNIMERGE	0.0	0.0	0.0	0.0
Gene Expression	0.0	0.0	0.0	0.0
MRI	2457.08	4397.99	1.0	11957.0

C – MRI

Domain	Average	Standard Deviation	Minimum	Maximum
ADNIMERGE	5.51	5.83	1.0	23.0
Gene Expression	1.55	0.94	1.0	7.0
MRI	2457.08	4397.99	1.0	11957.0

D - Total

Table S12 – CDR≥1.0 Subset

Table S13 – Resource Usage

Task	CPU Memory Per Job (Gigabytes)	GPU Memory Per Job (Gigabytes)	Compute Time Per Job (days:hours:minutes)	Disc Space Per Job (Gigabytes)	Number Of Jobs	Total Compute Time (days:hours:minutes)	Total Disc Space (Gigabytes)
**Getting The Known Values From ADNIMERGE**	3	*NA*	00:12:20	0.071	1	*NA*	*NA*
**Collecting The Medical Images Of Intersecting Patient IDs**	32	*NA*	00:01:02	20	1	*NA*	*NA*
**Converting The DICOM Images To PNG Format**	32	*NA*	00:12:25	7.9	1	*NA*	*NA*
**Processing The PNG Images And Converting Them To TXT Files**	64	*NA*	00:01:14	36	1	*NA*	*NA*
**Training The Autoencoders**	16	16	00:16:17	1.4	124	84:02:57	164
**Creating The MRI Domain**	128	16	00:01:53	11.015	1	*NA*	*NA*
**Completing The ADNIMERGE Data Domain**	64	*NA*	00:00:24	0.014	1	*NA*	*NA*
**Combining The Three Domains Into The Final Data Set**	1600	*NA*	00:03:26	12.016	1	*NA*	*NA*
**Correlation Analysis On The Entire Data Set**	128	*NA*	00:08:53	0.4051	6,418	2375:13:13	2,600
**Equalize The Size Of The Files Containing The Comparisons**	128	*NA*	05:10:25	2,600	1	*NA*	*NA*
**Getting The Significant Comparison Frequencies For Features By Bonferroni Alpha**	1600	*NA*	02:18:19	0.04	1	*NA*	*NA*
**Creating The Significant Comparison Frequencies Table For Bonferroni Alpha**	16	*NA*	01:10:37	0.027	1	*NA*	*NA*
**Filtering Comparisons By Maximum Alpha**	16	*NA*	00:00:08	0.0049	853	4:15:57	4.2
**Re-Run Only The Most Significant Comparisons On The Subsets**	16	*NA*	00:00:07	0.0014	14,780	66:22:33	21
